# Symmetrical Design of Biphenazine Derivative Anode for Proton Ion Batteries with High Voltage and Long‐Term Cycle Stability

**DOI:** 10.1002/advs.202401314

**Published:** 2024-06-14

**Authors:** Caixing Wang, Dunyong He, Huaizhu Wang, Jiandong Guo, Zhuoheng Bao, Yuge Feng, Linfeng Hu, Chenxi Zheng, Mengfan Zhao, Xuemei Wang, Yanrong Wang

**Affiliations:** ^1^ Institute of Innovation Materials and Energy School of Chemistry and Chemical Engineering Yangzhou University Yangzhou Jiangsu 225002 China; ^2^ School of Chemistry and Chemical Engineering Nanjing University Nanjing Jiangsu 210023 China; ^3^ School of Materials Science and Engineering Southeast University Nanjing Jiangsu 211189 China

**Keywords:** proton ion batteries, long‐term cycle stability, MnO_2_@graphite felt cathode, PbO_2_ cathode, phenazine derivative anodes

## Abstract

Organic anodes have emerged as a promising energy storage medium in proton ion batteries (PrIBs) due to their ability to reversibly accommodate non‐metallic proton ions. Nevertheless, the currently available organic electrodes often encounter dissolution issues, leading to a decrease in long‐cycle stability. In addition, the inherent potential of the organic anode is generally relatively high, resulting in low cell voltage of assembled PrIBs (<1.0 V). To address these challenges, a novel long‐period stable, low redox potential biphenylzine derivative, [2,2′‐biphenazine]‐7,7′‐tetraol (BPZT) is explored, from the perspective of molecular symmetry and solubility, in conjunction with the effect of the molecular frontier orbital energy levels on its redox potential. Specifically, BPZT exhibited a low potential of 0.29 V (vs SHE) and is virtually insoluble in 2 m H_2_SO_4_ electrolyte during cycling. When paired with MnO_2_@GF or PbO_2_ cathodes, the resulting PrIBs achieve cell voltages of 1.07 V or 1.44 V, respectively, and maintain a high capacity retention of 90% over 20000 cycles. Additionally, these full batteries can operate stably at a high mass loading of 10 mg_BPZT_ cm^−2^, highlighting their potential toward long‐term energy storage applications.

## Graphical abstract

Two phenazine derivatives are designed to reversibly accommodate protons. Experimental results show that structural symmetric [2,2′‐biphenazine]‐7,7′‐tetraol (BPZT) outperforms 2,3‐dihydrohyphenazine. BPZT exhibits a low potential of 0.29 V (vs SHE) with negligible dissolution during cycling. When paired with MnO_2_@GF or PbO_2_ cathodes, full batteries achieve high voltages of 1.07 V or 1.44 V, and maintain a high capacity retention over 20 000 cycles.

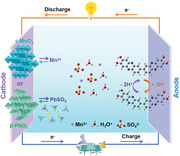

## 1 Introduction

The widespread adoption of clean energy sources such as solar and wind power is crucial for achieving carbon neutrality and promoting the development of green and sustainable energy. However, the intermittent nature of these renewable energy sources poses a challenge that needs to be addressed through large‐scale energy storage systems capable of peak shaving and valley filling.^[^ ^1^, ^2^ ^]^ Aqueous rechargeable batteries have emerged as promising candidates for large‐scale energy storage, benefiting from the advantages of intrinsic safety and high ionic conductivity of aqueous electrolytes.^[^ ^3^, ^4^ ^]^ Among them, proton serving as a charge carrier possesses attractive properties, such as small hydrated ion size (0.282 nm), fast proton diffusion dynamics, and ubiquitous in nature, which makes proton ion batteries (PrIBs) an appealing option for large‐scale energy storage applications.^[^ ^5^, ^6^, ^7^, ^8^, ^9^, ^10^ ^]^ In particular, traditional lead‐acid batteries which store proton and Pb^2+^ at the cathode and anode sides, respectively, have been a huge success for over a century due to the abundance of low‐cost raw materials and non‐flammable water‐based electrolyte. However, the limited cycle life (<500 cycles) hinders their application in grid energy storage.^[^ ^11^ ^]^

Until now, several inorganic and organic type materials have been investigated for reversible storage of proton, including Prussian blue analogues^[^ ^12^ ^]^ MnO_2_,^[^ ^13^ ^]^ and WO_3_·0.6H_2_O,^[^ ^14^ ^]^ H_1.75_MoO_3_,^[^ ^15^ ^]^ MXene^[^ ^16^ ^]^). Compared with conventional inorganic materials, organic electrodes are very promising candidates for energy‐storage devices due to their low cost‐efficiency, customizable structures, and abundant redox‐active sites, and they have been extensively applied in various metallic and non‐metallic ion batteries.^[^ ^17^, ^18^, ^19^ ^]^ Currently, the available organic electrodes for storing protons are mainly focused on quinones and their derivatives.^[^ ^20^ ^]^ For instance, Honma's group introduced a PrIB delivering a cell voltage of 0.6 V by coupling a tetrachlorohydroquinone cathode with an anthraquinone anode.^[^ ^21^ ^]^ Pyrene‐4,5,9,10‐tetraone (PTO) anode endowed with a high theoretical specific capacity (409 mAh g^−1^) and a moderate redox potential (0.5 V vs SHE) was paired with MnO_2_ and PbO_2_ cathodes, respectively, resulting in PrIBs demonstrating cell voltages of ≈0.86 and 1.25 V, respectively.^[^ ^13^, ^22^ ^]^ More recently, tetramethylquinone (TMBQ) and dibenzo [b, i] thianthrene‐5, 7, 12, 14‐tetraone have also been employed as anodes in combination with MnO_2_ cathode, leading to PrIBs with cell voltages of 0.86 and 0.94 V.^[^ ^23^, ^24^ ^]^ However, most PrIBs based on organic anodes still face a suboptimal cell voltage (<1.0 V) and inferior long‐term capacity retention rate, which impedes their practical applications.

Rational design and manufacture of organic electrodes hold immense importance in enhancing their overall electrochemical performance.^[^ ^25^, ^26^, ^27^, ^28^ ^]^ Theoretical studies have pointed out that introducing electron donating/withdrawing groups can elevate/depress parent molecules’ unoccupied molecular orbital (LUMO) energy level, and a higher LUMO energy level indicates a lower reduction potential.^[^ ^29^, ^30^ ^]^ For instance, Chen's group conducted a theoretical study on several benzoquinone derivatives for PrIBs, and discovered that TMBQ exhibited the lowest reduction potential due to the strong electron‐donating effect of the methyl groups.^[^ ^24^ ^]^ When it comes to all quinones, anthraquinone derivatives generally possess higher LUMO energy levels, making them a promising class of low‐potential anodes for proton storage.^[^ ^31^, ^32^ ^]^ However, many anthraquinone derivatives suffered from dissolution problems in the acidic electrolyte during prolonged cycling,^[^ ^20^ ^]^ which is unfavorable for long‐term energy storage.

In this work, we have utilized molecular engineering to rationally design two novel biphenazine derivatives, 2,3‐dihydrohyphenazine (DHP) and [2,2′‐biphenazine]−7,7′‐tetraol (BPZT), for the reversible storage of proton as anodes. In terms of molecular structure, BPZT consists of two DHP molecules connected by a single C─C bond, with a more symmetrical structure than the latter. In situ UV–vis spectra results indicate that BPZT with larger molecular size and more symmetric structure is almost insoluble in 2 m H_2_SO_4_ electrolyte compared with DHP, and thus delivering excellent long‐term cycling stability. Consequently, this work mainly focuses on the proton storage behavior in BPZT. The BPZT electrode exhibited a low redox potential of 0.29 V (vs SHE) in 2 m H_2_SO_4_ electrolyte. The C═N groups on the phenazine core store H^+^ ions, which are reversibly transformed between C─N─H. Moreover, the theoretical calculations support a symmetrical two‐proton and two‐electrons transfer pathway on the C═N groups in BPZT molecule. To demonstrate the feasibility of utilizing BPZT as an anode for H^+^ storage, two types of PrIBs were assembled by coupling with MnO_2_@GF or PbO_2_ cathodes. The two full batteries demonstrated high cell voltages of 1.07 and 1.44 V, and maintained a long‐term cycling stability with an excellent capacity retention rate of 90% over 20 000 cycles at 5 A g_BPZT_^−1^, outperforming most previously reported PrIBs. This work may provide valuable insights for the structural design of organic electrodes in energy storage devices.

## 2 Results and Discussion

**Figure 1:**
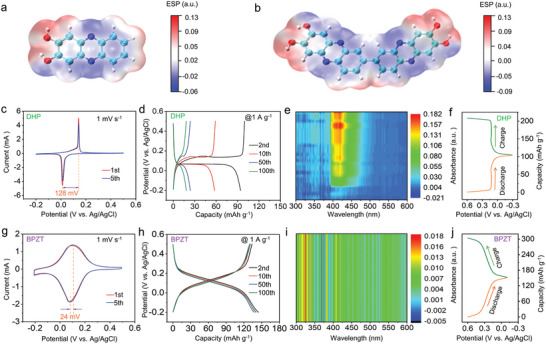
Comparison of calculated ESP distribution and electrochemical behaviors of DHP and BPZT in 2 m H_2_SO_4_ electrolyte. a,b) ESP distribution of DHP and BPZT molecules, with blue and red representing the electron‐rich and electron‐deficit regions within a molecule, respectively. c,g) CV curves of DHP and BPZT electrodes at the scan rate of 1 mV s^−1^ in the first and fifth cycles, respectively. d,h) Discharge/charge profiles of DHP and BPZT electrodes at a current density of 1 A g^−1^ at the 2nd, 10th, 50th and 100th cycles, respectively. e,i) The in situ UV–vis spectra of cycled electrolyte in the 20th cycle and f,j) the corresponding discharge‐charge curve of DHP and BPZT electrodes, respectively.

Both DHP and BPZT samples were synthesized through a straightforward cyclocondensation reaction of *o*‐phenylenediamine/2,5‐dihydroxybenquinone with 3,3′‐diaminobenzidine under mild conditions. The synthesis pathways of the two samples are illustrated in Scheme S1a,b (Supporting Information), following a previously reported work on multi‐hydroxyl phenazine derivatives.^[^ ^33^ ^]^ The structures of DHP and BPZT samples were first investigated by ^1^H nuclear magnetic resonance (^1^H‐NMR) spectra (Figures S1 and S2, Supporting Information), respectively. For DHP, the three strong peaks observed at chemical shifts ranging from δ 8.05 to 7.22 are related to three types of H atoms on the benzene core, with a peak area ratio of 1:1:1. In comparison, the relevant chemical shifts observed from δ 8.53 to 7.30 for BPZT are assigned to four types of H atoms on the benzene core, with a peak area ratio of 2:1:1:1. Besides, both samples show a similar chemical shift of hydroxyl groups at ≈ δ11. To further elucidate the molecular structure of the two samples, high‐resolution mass spectrometry was performed. As shown in Figures S3 and S4 (Supporting Information), m/z at 213.0657 ([M+H]^+^ peak) were found for DHP (calcd [M+H]^+^ 213.0664), which corresponded to an empirical formula of C_12_H_8_N_2_O_2_, and m/z at 423.1153 ([M+H]^−^ peak) were found for BPZT (calcd [M+H]^−^ 423.1093), which corresponded to an empirical formula of C_24_H_14_N_4_O_4_. Besides, the two samples show the same C═N vibration absorption peak at 1633 cm^−1^ according to their Fourier transform infrared (FT‐IR) spectra in Figures S5 and S6 (Supporting Information), which were not observed in the infrared spectra of their precursors (Figure S7, Supporting Information). Based on the above facts, it is confirmed that the structure of BPZT is very similar to DHP, but the number of functional groups has doubled. The scanning electron microscope (SEM) images (Figure S8, Supporting Information) revealed that DHP samples exhibited an irregular lumps, while the BPZT showed smaller particles with sizes ranging from ≈70 to ≈120 nm. To understand the spatial distribution of electrostatic charges around two molecules, we calculated their electrostatic potential (ESP) maps by density functional theory (DFT). Since the ESP usually refers to the electrostatic force exerted by the charge distribution within a molecule on a unit positive charge at a specific point in space, the value of the electrostatic potential around a molecule can be calculated to determine the reactive sites. As shown in **Figure** **1**a,b, the negative ESP region (represented by blue colors) indicates higher electronegativity and prefer electrophilic reactions, whereas the positive ESP region (represented by red colors) stands for nucleophilic center. Previous studies have shown that the solubility of organics in solution is correlated with the symmetry of molecular structure. Higher symmetrical molecules signifies lower solubility than lower symmetrical analogs according to Carnelley's rule.^[^ ^34^ ^]^ Because the symmetrical molecules can be more tightly and efficiently packed in the solid lattice, more energy is required to break the strong intermolecular forces, resulting in higher melting point and lower solubility. From the ESP results, it can be seen that the positive and negative ESP maps on the molecular surface of BPZT are more symmetrical compared to DHP, implying lower solubility of BPZT. On the other hand, the larger molecular size of BPZT is also beneficial to its low solubility in aqueous electrolytes. The electrochemical behavior of proton storage in DHP and BPZT electrodes was then compared. Cyclic voltammetry (CV) analysis of the DHP electrode (Figure 1c) revealed the presence of one pair of redox peaks at 0.015 V/0.143 V (vs Ag/AgCl) in 2 m H_2_SO_4_ electrolyte. The following half‐cell of DHP electrode measured at 1 A g^−1^ shows that DHP experienced a quick discharge capacity fade from 94.3 to 18.4 mAh g^−1^ during cycling from the 2nd to the 100th cycle (Figure 1d; Figure S9, Supporting Information). To discover the capacity decay mechanism, the in situ UV–vis spectroscopic measurement of the electrolyte was performed, which showed that the absorbance of the solution around λ_400 nm_ to λ_425 nm_ was continuously enhanced when the DHP was cycled (Figure 1e). Based on the observed absorption peak at λ_411 nm_ for 0.05 mm DHP in 2 m H_2_SO_4_ solution (Figure S10, Supporting Information), it can be concluded that the DHP electrode suffers from serious dissolution issues during charging and discharging process. In comparison, BPZT electrode (Figure 1g) also exhibits a reversible pair of redox peaks at 0.081/0.105 V (vs Ag/AgCl), but the peak gap (24 mV) was much narrower than the DHP electrode (128 mV). The average redox potential of BPZT electrode is 0.09 V vs Ag/AgCl (i.e., 0.29 V vs SHE), which is attractive among organic electrodes for proton storage (Table S1, Supporting Information). More importantly, the BPZT electrode only lost a minimal capacity of 5 mAh g^−1^ (from 145 to 140 mAh g^−1^) from the 2nd to the 100th cycle (Figure 1h) compared to the huge capacity loss of the DHP electrode. In situ UV–vis spectroscopic measurement of the relevant electrolyte reveals a negligible change in absorption intensity when BPZT electrode was cycled (Figure 1i), implying that BPZT electrode is almost insoluble in 2 m H_2_SO_4_ electrolyte during cycling. The extremely low solubility of BPZT electrode significantly enhances its electrochemical cycling stability, which will be further validated in subsequent long‐term cycle stability tests. In view of the much lower solubility of BPZT than DHP in 2 m H_2_SO_4_ electrolyte, we conducted a systematic study on the electrochemical performance of BPZT electrodes in the acidic electrolyte.

**Figure 2:**
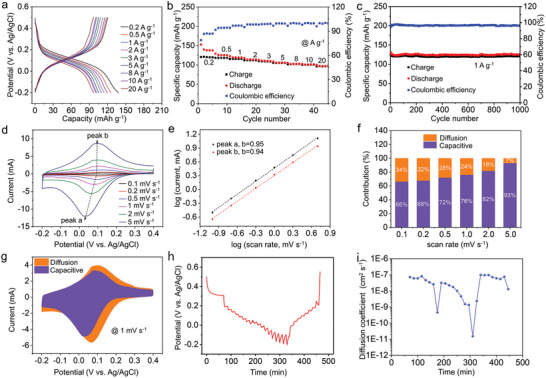
a) GCD curves of BPZT anode in a typical three‐electrode system in 2 m H_2_SO_4_ electrolyte at various current densities. b) The rate capability of BPZT anode at different current densities ranging from 0.2 to 20 A g^−1^. c) Cycle stability of BPZT anode at 1 A g^−1^. d) CV curves of BPZT anode at different scan rates ranging from 0.1 to 5 mV s^−1^. e) The relationship between log(*i*) and log(*v*) plots of anodic peak (peak a) and cathodic peaks (peak b) derived from the CV curves shown in Figure 1d. f) Diffusion and capacitive contribution at different scan rates from 0.1 to 5.0 mV s^−1^. g) CV curves and the capacitive contribution at 1 mV s^−1^. h) The GITT curves of BPZT electrode at 0.1 A g^−1^ and i) The H^+^ diffusion coefficient of BPZT electrode during a redox process.

The electrochemical performances of BPZT electrode in 2 m H_2_SO_4_ were characterized electrolyte in a typical three‐electrode system. **Figure** **2**a,b presents the rate performance of BPZT electrode at different current densities ranging from 0.2 to 20 A g^−1^. From the galvanostatic discharge/charge (GCD) profiles in Figure 2a, one pair of sloping plateaus can be observed mainly at the potential range of 0–0.2 V (vs Ag/AgCl). The BPZT electrode exhibits discharge and charge capacities of 138 and 120 mAh g^−1^ at 0.2 A g^−1^, with a Coulombic efficiency of 87%, which is related to hydrogen evolution. It is noteworthy that the achieved charge capacity (120 mAh g^−1^) is ≈50% of the theoretical capacity of 254 mAh g^−1^ (calculated based on four C═N bonds). The BPZT electrode demonstrates exceptional performance even at a high current density of 20 A g^−1^, which maintains a discharge capacity of 97 mAh g^−1^ with a high Coulombic efficiency of approaching 100%. These results indicate excellent rate capability and fast reaction kinetics of BPZT electrode. Figure 2c shows the cycling performance of BPZT electrode at 1 A g^−1^, and the discharge capacity decline from 132 to 126 mAh g^−1^ over 1000 cycles, with a capacity retention of 95%. In order to study the effect of concentration of H_2_SO_4_ on the electrochemical stability of the BPZT electrode, we tested the cycling performance of the electrode in 0.5 m and 4 m H_2_SO_4_ electrolytes, respectively, and the results showed that the BPZT electrode exhibited excellent cycling stability in both electrolytes (Figure S11, Supporting Information). Figure 2d depicts the CV curves of BPZT electrode obtained at different scan rates ranging from 0.1 to 5 mV s^−1^. The current (*i*) responses versus scan rates (*v*) obey a power‐law relationship, *i*_p_ = *av^b^*,^[^ ^35^ ^]^ in which *a* and *b* are the adjustable parameters. Figure 2e presents the correlation between log (*i*) and log (*ν*) of anodic peak (peak a) and cathodic peak (peak b). The *b*‐value determined by the slopes of the two peaks are 0.95 and 0.94, indicating the predominance of capacitive current. Furthermore, the current contribution proportion from the capacitance effect (*k*_1_ *v*) and diffusion process (*k*_2_ *v* ^1/2^) can be quantitatively calculated according to the equation, *i*(V) = *k*_1_ *v* + *k*_2_ *v* ^1/2^.^[^ ^36^ ^]^ As a result, the surface capacitive contribution boosts from 66% to 93% with the increase of scan rates from 0.1 to 5 mV s^−1^ (Figure 2f,g). The results signify the surface capacitive‐dominant charge storage nature and the slight diffusion‐limited process for the BPZT electrode. The Nyquist and bode plots of the BPZT electrode in 2 m H_2_SO_4_ electrolyte are provided, as shown in Figure S12 (Supporting Information). The results suggest the low resistance and rapid charge transfer in BPZT electrode, thus conducive to the fast reaction kinetics, which is consistent with the above analyses. In addition, the diffusion coefficient (D) of proton was measured by the galvanostatic intermittent titration technique (GITT), shown in Figure 2h,i. BPZT electrode is endowed with the D value ranging from 8.2 × 10^−8^ to 1.4 × 10^−9^ cm^2^ s^−1^ during the discharge process and 2.4 × 10^−9^ to 9.7 × 10^−8^ cm^2^ s^−1^ during the charge process, respectively.

**Figure 3:**
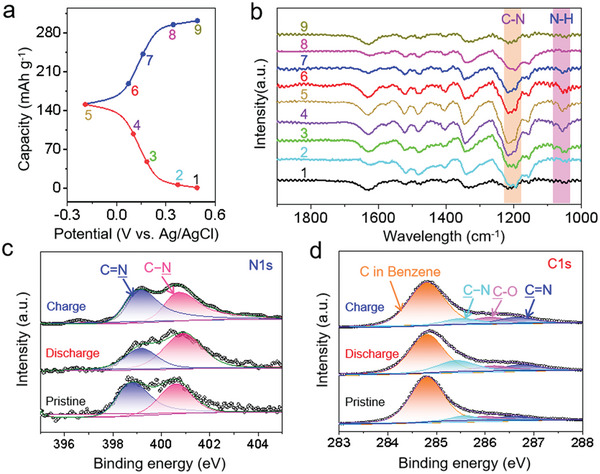
a) GCD curve at a current density of 0.1 A g^−1^ and b) corresponding ex situ FT‐IR spectra of BPZT electrode at different states. c,d) The N 1s and C 1s XPS spectra of BPZT electrode at different states, i) pristine, (ii) reduced state, (iii) oxidized state, respectively.

In order to elucidate the intrinsic energy storage mechanism of BPZT electrode in 2 m H_2_SO_4_ electrolyte, its structural evolution during discharge/charge processes was investigated by ex situ FT‐IR spectroscopy and X‐ray photoelectron spectroscopy (XPS), respectively. The GCD curve of BPZT electrode was recorded at a current density of 0.1 A g^−1^ (**Figure** **3**a), and the corresponding ex situ FT‐IR spectra of BPZT electrode at different states were shown in Figure 3b. The intensity of the adsorption peaks at 1183 cm^−1^ assigned to the C─N bond and 1069 cm^−1^ assigned to the N─H bond^[^ ^37^ ^]^ continuously increases upon discharge process, which are attributed to the conversion of the C═N groups to C─N─H groups. In the subsequent charge step, the peak intensities of these groups show a reverse tendency, indicating the reversible conversion between C─N groups and C═N groups. The survey XPS spectra on BPZT electrode at pristine, reduced, and re‐oxidized states were collected in Figure S13 (Supporting Information). When the electrode was discharged to −0.2 V (vs Ag/AgCl), the peak intensity of N 1s XPS spectra (Figure 3c) at 398.7 eV (C═N bond) weakens, while the peak intensity at 400.6 eV (C─N bond) strengthens.^[^ ^38^ ^]^ It should be noted that the peak associated with the C═N bond doesn't completely disappear, implying that only partial of C═N bonds can be transformed into C─N bonds. When recharged to 0.5 V (vs Ag/AgCl), the changes observed are opposite to the discharged state. In addition, the intensity change trend for C 1s spectra (Figure 3d) at ≈285.6 eV (C─N) and ≈286.6 eV (C═N) during the redox process (from I to II and III) is consistent with the N 1s spectra,^[^ ^39^ ^]^ further confirming the reversible transformation between C═N and C─N bonds. Otherwise, the peak at ≈284.8 eV is derived from C in benzene ring, and the minor peak at ≈286.0 eV is associated with C─O. By combining the ex suit FT‐IR, XPS results, and the achieved capacity, the energy storage mechanism is speculated to involve the reversible conversion of half of the C─N/C═N bonds in the BPZT electrode during the discharge/charge process. Additionally, the in situ electrochemical quartz crystal microbalance (EQCM) was carried out to determine the mass change of the electrode during a discharge process (Figure S14, Supporting Information). The Δm/Δq between 0.2 and 0.05 V (vs Ag/AgCl) is 3.0 g mol^−1^ of e^−1^, which is very close to the formula mass of H^+^ (1.0 g mol^−1^), indicating that the charge carrier introduced into the BPZT electrode is H^+^ rather than H_3_O^+^.

**Figure 4:**
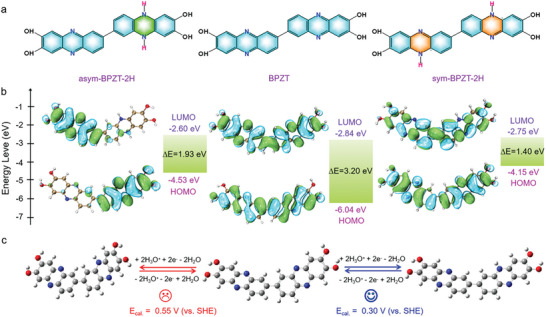
DFT calculation results about the reaction mechanism of BPZT. a) The molecular structures of BPZT and possible reduction products asym‐BPZT‐2H and sym‐BPZT‐2H. b) Calculated frontier molecular orbital energy levels of BPZT and BPZT‐2H molecules, respectively. c) Schematic diagram of two possible redox reaction pathways of BPZT based on a two‐electron reaction process.

To better understand the reaction mechanism of the BPZT electrode, DFT calculations were conducted to determine the optimized structures and the Gibbs free energy of BPZT molecule and its possible reduced forms. **Figure** **4**a shows the molecular structures of BPZT and two‐electron reduction products asym‐BPZT‐2H or sym‐BPZT‐2H. From the molecular electrostatic potential (ESP) mapping of BPZT (Figure 1a), asym‐BPZT‐2H and sym‐BPZT‐2H (Figure S15, Supporting Information), it is observed that the negative potential area of C═N groups is the potential reactive sites of BPZT molecule for storing protons, agreed with the *ex‐situ* FT‐IR and XPS results in Figure 3. Frontier molecular orbital energy level analyses of BPZT and asym ^−1^sym‐BPZT‐2H molecules reveal that the HOMO and LUMO energy gap of sym‐BPZT‐2H is narrower than that of asym‐BPZT‐2H. To gain insight into which redox process is more reasonable (Figure 4c), the redox potential of BPZT via the two routes mentioned above were also evaluated based on the Gibbs free energy change of different states (Tables S2 and S3, Supporting Information). The calculated redox potential of BPZT/sys‐BPZT‐2H is 0.30 V (vs SHE), very close to the experimental value of 0.29 V (vs SHE). In comparison, the calculated redox potential of BPZT/asym‐BPZT‐2H is high up to 0.55 V (vs SHE), which significantly deviates from the experimental value. To confirm the reasonableness of the calculations even further, the redox potential predictions for the one‐step three/four‐electron reduction of BPZT were also calculated. The optimized structures of BPZT‐3H and BPZT‐4H with their ESP and energy levels are shown in Figure S16 (Supporting Information). Based on the Gibbs free energy change of BPZT and BPZT‐3H or BPZT‐4H, the predicted redox potentials of BPZT/BPZT‐3H and BPZT/BPZT‐4H are 0.47 V (vs SHE) and 0.53 V (vs SHE), respectively (Figure S17, Supporting Information), which are also far from the experimental result, suggesting improbable processes for BPZT. Therefore, we speculate that the fact that the BPZT (Figure 2c) exhibited about half of the theoretical specific capacity is related to the more stable structure of sys‐BPZT‐2H after semi‐reduction of BPZT, which prevents the whole molecular system from further electron enrichment.

**Figure 5:**
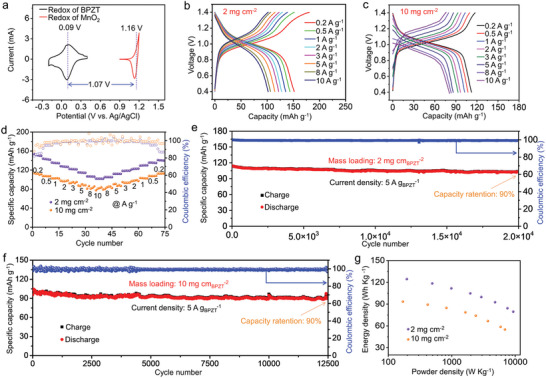
Electrochemical performances of the MnO_2_@GF//BPZT full battery. a) CV profiles of BPZT anode and MnO_2_@GF cathode at 1 mV s^−1^, respectively. b,c) GCD profiles and d) rate performance of the full battery at various current densities ranging from 0.2 to 10 A g_BPZT_^−1^ with mass loadings of 2 and 10 mg_BPZT_ cm^−2^, respectively. e,f) Long‐term cycling performance of the full battery at 5 A g_BPZT_^−1^ with mass loadings of 2 and 10 mg_BPZT_ cm^−2^. g) Energy density versus power density of the full battery with mass loadings of 2 and 10 mg cm^−2^, respectively.

To evaluate the potential application of BPZT anode, we assemble a full battery with electroplated MnO_2_ (ε‐phase) onto graphite felt (MnO_2_@GF) as the cathode. The morphology, structure, and electrochemical properties of electrodeposited MnO_2_ were analyzed using SEM, X‐ray diffractometer (XRD) pattern, XPS spectra, and GCD curves (Figures S18–S22, Supporting Information). The CV curves of BPZT anode and MnO_2_@GF cathode in a hybrid electrolyte of 2 m MnSO_4_ + 2 m H_2_SO_4_ are shown in **Figure** **5**a. Based on the CV diagram, the designed full battery was expected to show a decent cell voltage of 1.07 V. The cycling performance of MnO_2_@GF//BPZT cell at a low current density of 0.05 A g^−1^ was measured (Figure S23, Supporting Information). The result showed that the discharge capacity was maintained at ≈ 135 mAh g_BPZT_^−1^ over 40 cycles despite the undesirable Coulombic efficiency, which confirmed the good stability of BPZT electrode in the hybrid electrolyte. The rate performance of the full battery was then evaluated at different mass loadings of the BPZT anode, presented in Figure 5b–d, respectively. At a mass loading of 2 mg_BPZT_ cm^−2^, the MnO_2_@GF//BPZT full battery delivers a specific capacity of 152 mAh g_BPZT_^−1^ at 0.2 A g_BPZT_^−1^, and it maintains 86 mAh g_BPZT_^−1^ at an ultrahigh current density of 20 A g_BPZT_^−1^. Even increasing the mass loading to 10 mg_BPZT_ cm^−2^, the capacity still could achieve to 112 mAh g_BPZT_^−1^ at 0.2 A g^−1^ and 81 mAh g_BPZT_^−1^ at 20 A g_BPZT_^−1^, respectively. Additionally, the MnO_2_@GF//BPZT full batteries demonstrate robust long‐term cycling stability. The cell with an anodic mass loading of 2 mg_BPZT_ cm^−2^ shows a very slow capacity decay from 114 to 102 mAh g_BPZT_^−1^ over 20 000 cycles at 5 A g_BPZT_^−1^ (Figure 5e), and the cell maintains a capacity retention rate of 90% over 12 500 cycles (Figure 5f) even increasing the anodic mass loading to 10 mg_BPZT_ cm^−2^. The energy density and power density of the full cell are calculated based on the total active mass of the anode and assumed cathode materials according to the data in Figure 5b,c. At a mass loading of 2 mg_BPZT_ cm^−2^, the cell achieves a maximum energy density of 125 Wh kg^−1^ at a power density of 196 W kg^−1^ and maintained 80 Wh kg^−1^ at 9320 W kg^−1^. Even at a high mass loading of 10 mg_BPZT_ cm^−2^, the cell delivers an energy density of 94 Wh kg^−1^ at a power density of 169 W kg^−1^ and maintains 55 Wh kg^−1^ at 6885 W kg^−1^ (Figure 5g). The excellent electrochemical performance is attributed to the low resistance of the full battery (Figure S24, Supporting Information). In addition, self‐discharge test of MnO_2_//BPZT cell were conducted (Figure S25, Supporting Information). It can be observed the charge capacity is ≈ 130 mAh g^−1^, and after 6, 12, and 24 h rest, the discharge capacity is slightly decreased from 129.3 to 125.5 mAh. The capacity loss rate (91.4% after 24 h) is slightly higher than that of PTO (98% after 24 h).^[^ ^13^ ^]^

**Figure 6:**
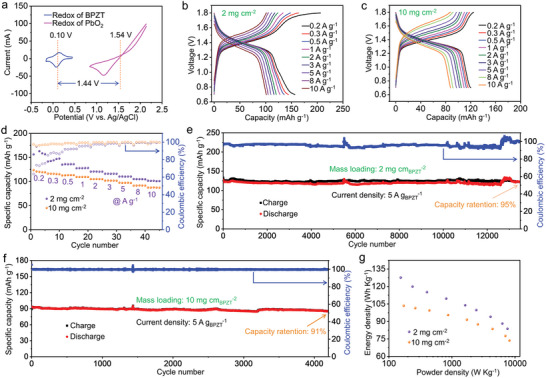
Electrochemical performances of the PbO_2_//BPZT full battery. a) CV profiles of BPZT anode and PbO_2_ cathode at 1 mV s^−1^, respectively. b,c) GCD profiles and (d) Rate performance of the full battery at various current densities range from 0.2 to 10 A g_BPZT_^−1^ with mass loadings of 2 and 10 mg_BPZT_ cm^−2^, respectively. e,f) Long‐term cycling performance of the full battery at 5 A g_BPZT_^−1^ with mass loadings of 2 and 10 mg_BPZT_ cm^−2^. g) Energy density versus power density of the full battery with mass loadings of 2 and 10 mg_BPZT_ cm^−2^, respectively.

To ensure compatibility with the power demands of modern devices, it is crucial to effectively enhance the voltage output of batteries. With this objective in view, the PbO_2_//BPZT battery has been developed to assess the merits of the battery system by utilizing a low‐cost commercial PbO_2_ cathode with higher potential in comparison to MnO_2_@GF cathode. The morphology, structure and electrochemical properties of PbO_2_ cathode were characterized by SEM images, XRD pattern, and GCD curves (Figures S26–S28, Supporting Information). The CV curves depicted in **Figure** **6**a reveal that the redox potentials of BPZT anode and PbO_2_ cathode in 4 m H_2_SO_4_ electrolyte are 0.10 V (vs Ag/AgCl) and 1.54 V (vs Ag/AgCl), respectively, predicting an average cell voltage of 1.44 V, which surpasses the majority of reported PrIBs based on organic electrodes (Table S4, Supporting Information). The reason we use 4 m H_2_SO_4_ electrolyte is that PbO_2_ cathode is not stable in 2 m H_2_SO_4_ (Figure S29, Supporting Information), resulting the fast capacity decay of PbO_2_//BPZT full battery (Figure S30, Supporting Information). The cycling performance of PbO_2_//BPZT full battery at a low current density of 0.05 A g^−1^ was tested (Figure S31, Supporting Information). The results showed that the discharge capacity was stably kept ≈ 145 mAh g_BPZT_^−1^ over 40 cycles despite of the relatively low Coulombic efficiency. Then, the rate performance of the PbO_2_//BPZT full cell was evaluated at different mass loadings of BPZT anode, respectively (Figure 6b–d). At a mass loading of 2 mg_BPZT_ cm^−2^, the cell exhibits a specific capacity of 156 mAh g^−1^ at 0.2 A g_BPZT_^−1^ and maintains a specific capacity of 102 mAh g^−1^ at 10 A g_BPZT_^−1^. The full cell demonstrates excellent rate capability even at a high mass loading of 10 mg_BPZT_ cm^−2^. Furthermore, the cell delivers a long‐term cycle stability. At a mass loading of 2 mg_BPZT_ cm^−2^, the cell undergoes over 13 500 cycles at 5 A g_BPZT_^−1^, with a capacity retention rate of 95% (Figure 6e). Even at a high mass loading of 10 mg_BPZT_ cm^−2^, the full cell still maintains its stability over 4200 cycles at 5 A g_BPZT_^−1^, with a capacity retention rate of 91% (Figure 6f). Additionally, the energy density and power density of the full cell are calculated based on the total mass of the BPZT anode and consumed PbO_2_ cathode, presented in Figure 6g. At a mass loading of 2 mg_BPZT_ cm^−2^, the full battery possesses a maximum energy density of 128 Wh kg^−1^ at a power density of 154 W kg^−1^. At a high power density of 7612 W kg^−1^, the energy density retains 84 Wh kg^−1^. At a high mass loading of 10 mg_BPZT_ cm^−2^, the full cell delivers an energy density of 103 Wh kg^−1^ at a power density of 172 W kg^−1^ and maintains an energy density of 74 Wh kg^−1^ at a power density of 8180 W kg^−1^. The Nyquist plots of PbO_2_//BPZT full cell was displayed in Figure S32 (Supporting Information). The low resistance value supports the outstanding rate performances of the PbO_2_//BPZT full battery. In comparison, DHP with asymmetric structure as anode was also integrated with MnO_2_@GF or PbO_2_ cathodes, and these constructed full battery systems exhibited noteworthy degradation in cycling performance (Figure S33, Supporting Information), which is significantly inferior to full batteries based on BPZT anode. Of course, compared to other aqueous batteries such as zinc‐ion or ammonium‐ion batteries, the designed PrIBs utilize highly corrosive H_2_SO_4_ electrolyte. Consequently, it is imperative to rigorously prevent electrolyte leakage to safeguard the environment from hazards. Additionally, overcharging must be avoided to mitigate the safety risks associated with hydrogen evolution side reactions.

## 3 Conclusion

In summary, we have rationally demonstrated two phenazine derivatives to serve as anodes for PrIBs. Compared to DHP, the BPZT molecule with larger molecular size and higher structural symmetry exhibited hardly dissolution issue in 2 m H_2_SO_4_ electrolyte, thus allowing for its long‐term sustainable operation. Additionally, the BPZT electrode exhibits low redox potential (0.29 V vs SHE) and undergoes a symmetric two‐electron redox reaction associated with the transformation of C═N/C─N during the reversible uptake/release of H_3_O^+^. When paired with MnO_2_@GF or PbO_2_ cathodes, the constructed PrIBs demonstrate cell voltages of 1.07 V or 1.44 V, and a high capacity retention of 90% over 20 000 cycles. Furthermore, these full batteries can operate stably at a high mass loading up to 10 mg_BPZT_ cm^−2^, standing out among most reported PrIBs based on organic electrodes. This work may be a useful insight for the structural design of other organic molecules for utilization in the field of energy storage.

## 4 Experimental Section

### Materials Preparation—Synthesis of DHP

Briefly, 10 mmol (1.08 g) *o*‐phenylenediamine and 10.5 mmol (1.47 g) 2,5‐dihydroxybenquinone were added into CH_3_COOH solution, heated at 40 °C and stirred for 12 h. The mixture was cooled down to room temperature and then filtrated, rinsed with H_2_O and ethanol for several times, respectively, and then dried under vacuum at 80 °C for 6 h.

### Synthesis of BPZT

Briefly, 10 mmol (2.14 g) 3,3′‐diaminobenzidine and 10.5 mmol (1.47 g) 2,5‐dihydroxybenquinone were added into CH_3_COOH solution, heated at 40 °C and stirred for 12 h. The mixture was cooled down to room temperature and then filtrated, rinsed with H_2_O and ethanol for several times, respectively, and then dried under vacuum at 80 °C for 6 h.

### Synthesis of Electrodeposited MnO_2_ on GF

The MnO_2_ cathode was prepared by electrodeposition method onto a GF electrode. A typical three‐electrode system was employed with Ag/AgCl electrode as reference electrode, graphite rod as counter electrode, and commercial GF as working electrode in the acid electrolyte composed by 2 m MnSO_4_ + 2 m H_2_SO_4_. The three‐electrode system was galvanostatic charged at 1 mA cm^−2^ to 2 V for different electrochemical deposition time. The as‐obtained MnO_2_@GF was washed with deionized water and ethanol, respectively, and then dried in a vacuum oven at 70 °C for 2 h.

### Preparation of PbO_2_

PbO_2_ cathodes were obtained by dismantling from discarded lead‐acid batteries (Tianneng Battery Group Co., Ltd).

### Characterization

^1^H‐NMR was conducted by a 500 MHz NMR spectrometer (AVANCE III HD) to define the structure of the BPZT sample. High‐resolution mass spectra (HRMS) were examined by Electrospray ionization mass spectrometry (Bruker Dalton, maXis). FT‐IR spectra were measured using Thermo Fisher Nicolet 6700 FT‐IR spectrometer to characterize the functional groups of DHP and BPZT samples and relevant electrodes at different discharge‐charge states. In particular, in situ UV–vis spectroscopic tests were performed on the DHP and BPZT electrodes to monitor any dissolution during the discharge‐charge process. A small three‐electrode system was constructed in a cuvette using DHP or BPZT as the working electrode, platinum wire as the counter electrode and Ag/AgCl as the reference electrode. Discharge‐charge tests were performed using a DH7001 electrochemical workstation together with continuous real‐time UV spectroscopy. The morphology and microstructure of the sample was characterized by SEM (Field‐emission JEOL JSM‐6390). The surface elements and electronic states were characterized via XPS (PHI 5000C&PHI5300). X‐ray diffractometer (XRD, Bruker D8 Advance, Germany) with Cu K*α* radiation (λ = 0.15 406 nm) was used to analyze the structures MnO_2_@GF cathode.

### Electrode Preparation and Battery Fabrication

BPZT electrodes were prepared by mixing 60 wt.% of BPZT, 30 wt.% of conductive additive (Ketjen black, ≥99.9%, Sinopharm), and 10 wt.% of binder (polytetrafluorethylene (PTFE), Sigma‐Aldrich) in ethanol (≥99.7%, Sigma–Aldrich) solvent. The slurry was rolled out into a thin film, and dried at 60 °C for 10 h in a vacuum oven, and then the thin film was compressed onto titanium mesh. The typical active organic material mass loading was 2 mg_BPZT_ cm^−2^, except for 10 mg_BPZT_ cm^−2^ in high mass loading performance testing. The electrochemical performance of DHP or BPZT electrodes was evaluated using a typical three‐electrode system, in which graphite rod and Ag/AgCl electrode were served as the counter electrode and reference electrode, respectively, with 2 m or 4 m H_2_SO_4_ aqueous solution as electrolyte. The electrolyte utilized in the full battery system with MnO_2_@GF as the cathode was a hybrid electrolyte composed of 2 m H_2_SO_4_ + 2 m MnSO_4_. The electrolyte used in the full battery system with PbO_2_ as the cathode was 4 m H_2_SO_4_. Cyclic voltammetry (CV) tests were conducted on an electrochemical workstation (DH7001). The GCD measurements were performed on LAND‐CT3002A battery‐testing instrument. The electrochemical impedance spectroscopy (EIS) analyses of half battery and full battery were also conducted on an instrument of DH7001. The measuring frequency range ranges from 0.1 and 100 kHz.

In situ EQCM measurement was carried out in a three‐electrode battery using quartz microcrystals (QCM200, Stanford Research Systems, Inc.). BPZT, Super P, and polyvinylidene fluoride (PVDF) with a weight ratio of 8:1:1 were mixed in N‐methylpyrrolidone (NMP) with stirring. The slurry was loaded onto microcrystals by spraying and then dried at 80 °C for 12 h. The corresponding BPZT electrode was served as a working electrode. Ag/AgCl electrode and Pt electrode were employed as reference electrode and counter electrode, respectively. CV measurement was conducted by using an electrochemical workstation (Biologic SP‐150). The EQCM response correlates with the mass changes of the electrode due to ions and/or solvent molecules interactions according to the following Sauerbrey's Equation (1):

(1) $$\Delta f=-C_{f}\cdot \Delta m\;$$

where Δ*f* and Δ*m* represent frequency and mass change, respectively. C_f_ is the sensitivity factor for the crystal (56.6 Hz µg^−1^ cm^2^).

### Computational Details

All DFT calculations were performed by using the Gaussian 16 software package.^[^ ^40^ ^]^ The geometry optimizations and frequency calculations for the mentioned species were carried out using B3LYP‐D3(BJ) functionals with 6–311G(d,p) basis set. The implicit CPCM solvent model was used to represent the solvent effect of water molecules. Electrostatic potential mapping, LUMO, and HOMO graphics were obtained using Multiwfn^[^ ^41^ ^]^ and VMD.^[^ ^42^ ^]^ In order to calculate the redox potentials of several possible reaction pathways of BZPT, the Gibbs free energies of BPZT, asym‐BPZT‐2H, sym‐BPZT‐2H, BPZT‐3H, and BPZT‐4H in water were calculated and listed in Table S2 (Supporting Information). It has been reported that the proton hydration‐free energy is −262.4 kcal mol^−1^.^[^ ^43^ ^]^ The redox potential was calculated based on the Nernst equation, and the value was then converted to a potential relative to the standard hydrogen electrode (E_SHE_ = 4.28 V),^[^ ^44^ ^]^ as follows:

(2) $$E=-\Delta G/\mathrm{nF}-E_{\mathrm{SHE}}$$

where ΔG is the difference between the Gibbs free energies of products and reactants in the overall reaction, F is the Faraday constant equal to 1 *e*, and *n* is the number of electrons transferred. The calculated redox potentials of different redox processes are listed in Table S3 (Supporting Information).

## Conflict of Interest

The authors declare no conflict of interest.

## Author Contributions

C.X.W. performed the synthesis of materials, characterizations and wrote the original manuscript. D.Y.H performed the electrochemical measurements and contributed to the formal analysis. H.Z.W. performed the HRMS measurement. J.D.G. and C.X.Z. performed the DFT calculations. Z.H.B. and L.F.H. contributed to the EQCM test and analysis. Y.G.F. contributed to the Bode analysis. M.F.Z. performed the SEM. X.M.W. performed the XRD measurement. Y.R.W. supervised the study and wrote the manuscript.

## Supporting information

Supporting Information

## Data Availability

The data that support the findings of this study are available from the corresponding author upon reasonable request.
