# Supplementary material for: Symmetrical Design of Biphenazine Derivative Anode for Proton Ion Batteries with High Voltage and Long‐Term Cycle Stability
Source: Adv Sci (Weinh). 2024 Jun 14;11(30):2401314. doi: 10.1002/advs.202401314 (PMC11321615; doi:10.1002/advs.202401314)
Supplement: Supplementary file 1 — Supporting Information [file ADVS-11-2401314-s001.docx]

Supporting Information

Symmetrical design of biphenazine derivative anode for proton ion batteries with high voltage and long-term cycle stability

Caixing Wang, Dunyong He, Huaizhu Wang, Jiandong Guo, Zhuoheng Bao, Yuge Feng, Linfeng Hu, Chenxi Zheng, Mengfan Zhao, Xuemei Wang, and Yanrong Wang*


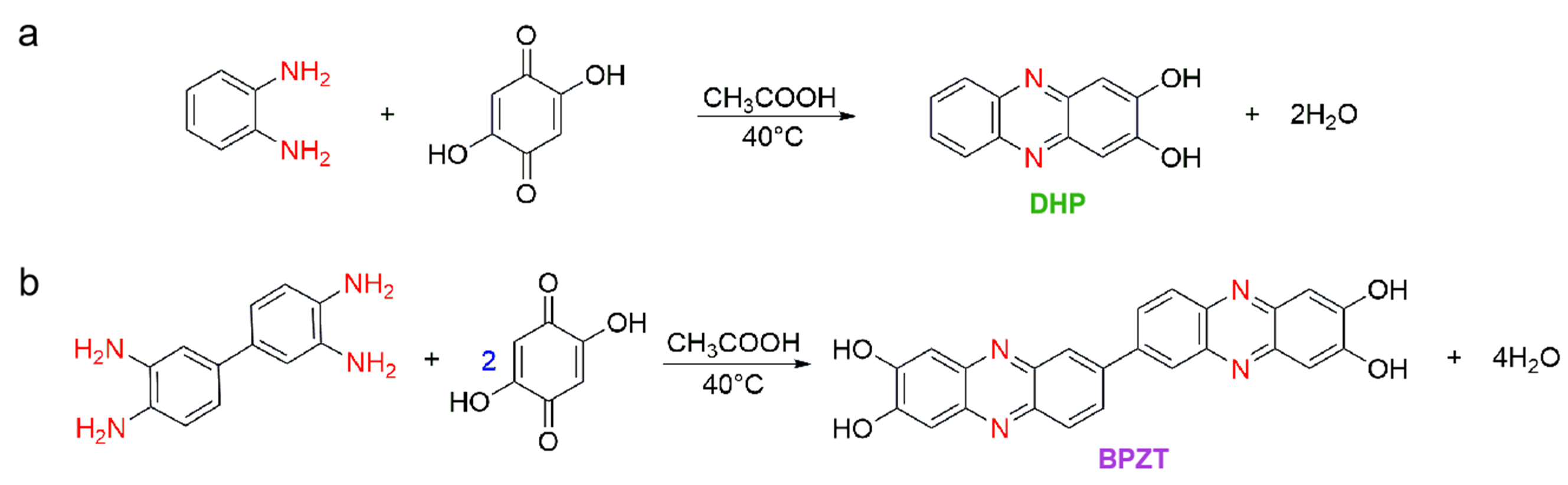


**Scheme S1**. Schematic synthesis routes of (a) DHP and (b) BPZT samples.


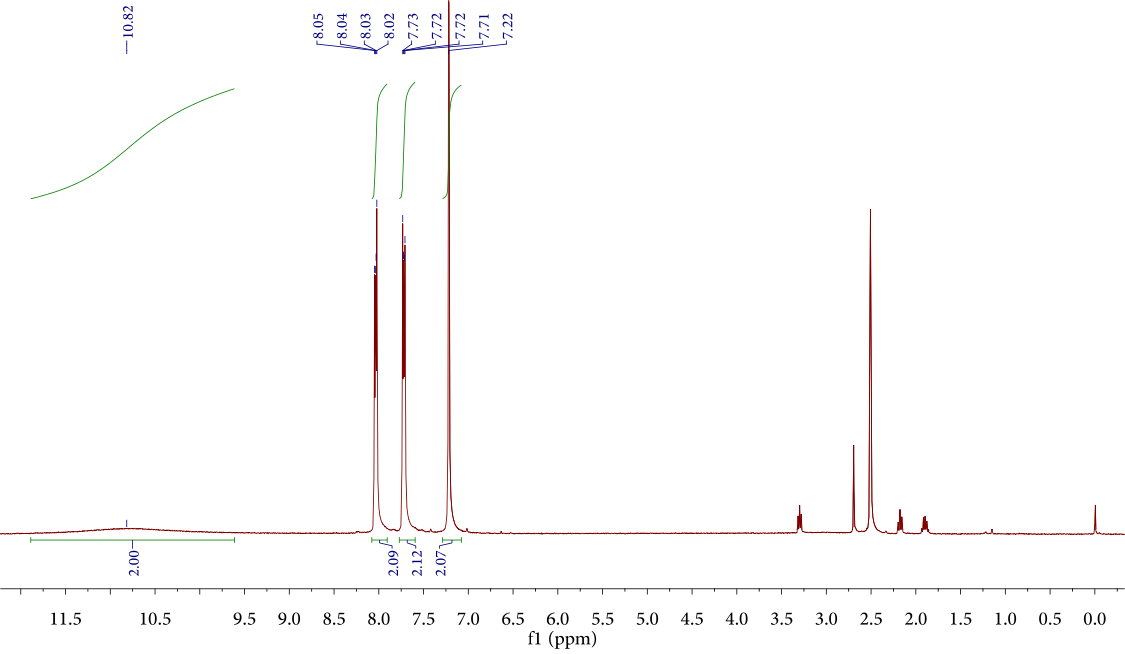


**Figure S1.** ^1^H NMR spectrum (500 MHz, DMSO-d6) of as-prepared DHP sample. δ 10.82 (s, 2H), 8.02~8.05 (dd, 2H), 7.71~7.73 (dd, 2H), 7.22 (s, 2H).

The three strong ^1^H NMR spectra at the chemical shifts ranging from δ 8.05 to 7.22 are related to three species of hydrogen in the benzene ring, with a peak area ratio of 1:1:1. The chemical shift of δ 10.82 belongs to the hydrogen on the phenol hydroxyl group.


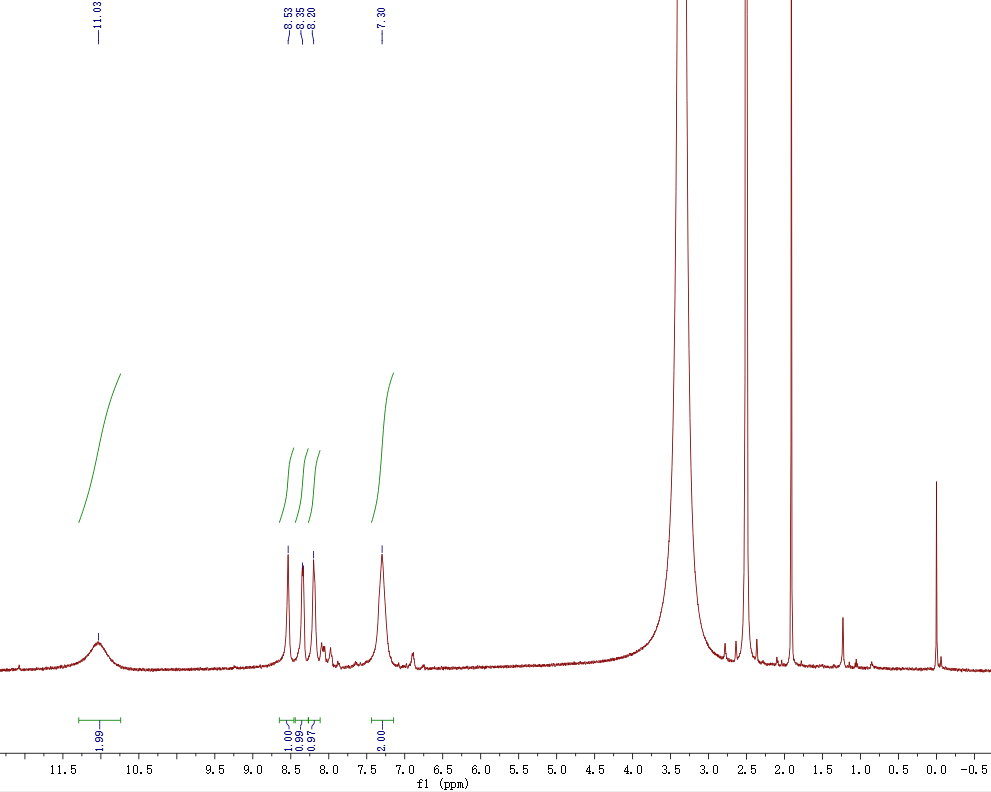


**Figure S2.** ^1^H NMR spectrum (500 MHz, DMSO-d6) of as-prepared BPZT sample. δ 11.03 (s, 4H), 8.53 (s, 2H), 8.35 (s, 2H), 8.20 (s, 2H), 7.30 (s, 4H).

The relevant chemical shifts ranging from δ 8.53 to 7.30 are observed in ^1^H NMR spectrum, which indicates the presence of four species of hydrogen in the benzene ring region, with a peak area ratio of 2:1:1:1. The chemical shift of δ 11.03 is attributed to the hydrogen on the phenol hydroxyl group.


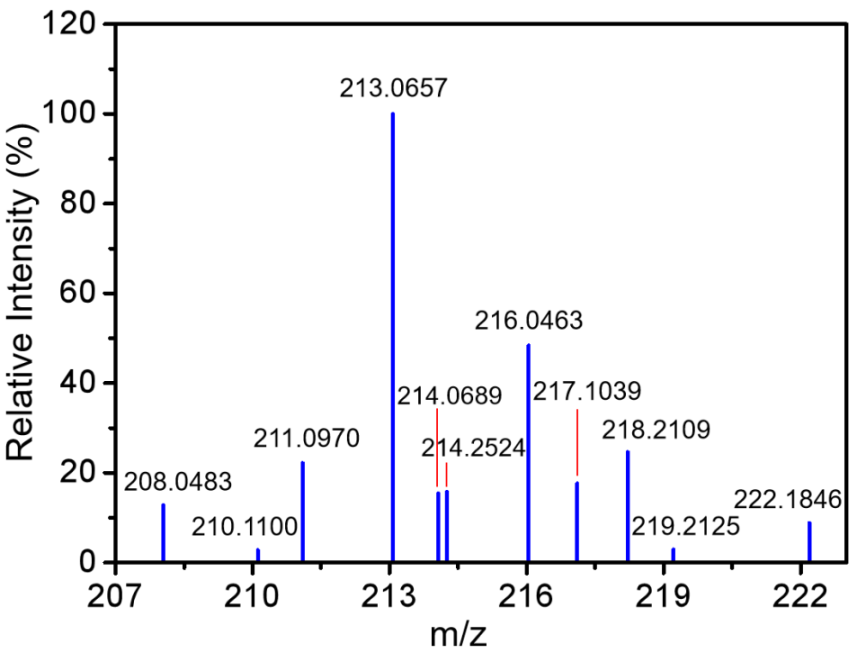


**Figure S3.** HRMS spectrum of DHP in a positive mode. The observed m/z peak at 213.0657 is assigined to [M+H]^+^ peak (calcd:213.0664).


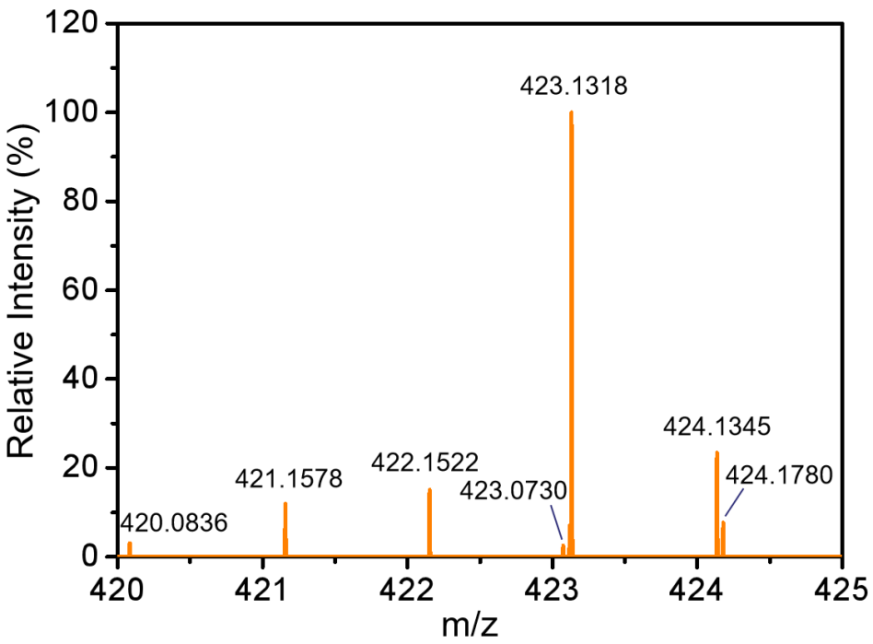


**Figure S4.** HRMS spectrum of BPZT in a negative mode. The observed m/z peak at 422.1153 is assigned to [M+H]^-^ peak (calcd:423.1093).


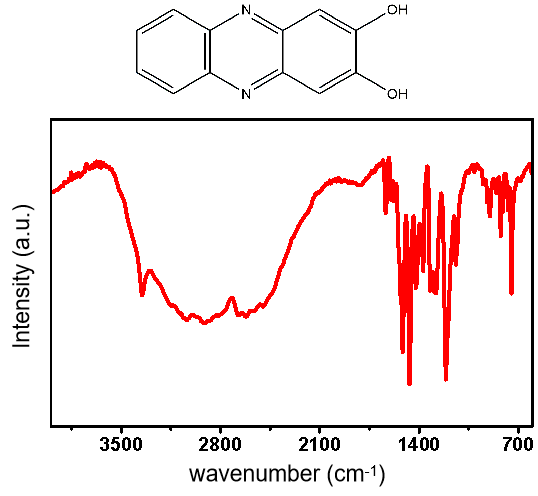


**Figure S5.** FT-IR spectra of DHP. The peaks at around 3347 cm^-1^ can be assigned to the stretching vibration of O-H bond. Specifically, the peak observed at 1633 cm^-1^ corresponds to the stretching vibration of C=N bonds. The peaks located at 1565, 1465 and 1419 cm^-1^ can be attributed to C=C bonds in skeleton vibration of aromatic rings. The peak at 1371 cm^-1^ corresponds to the stretching vibration of C-N bond. The peak located at 1200 cm^-1^ matches with stretching vibration of C-O bond. The peaks located at 900, 825 and 742 cm^-1^ attach to the in-plane and out-of-plane bending vibrations of C-H bond, respectively.


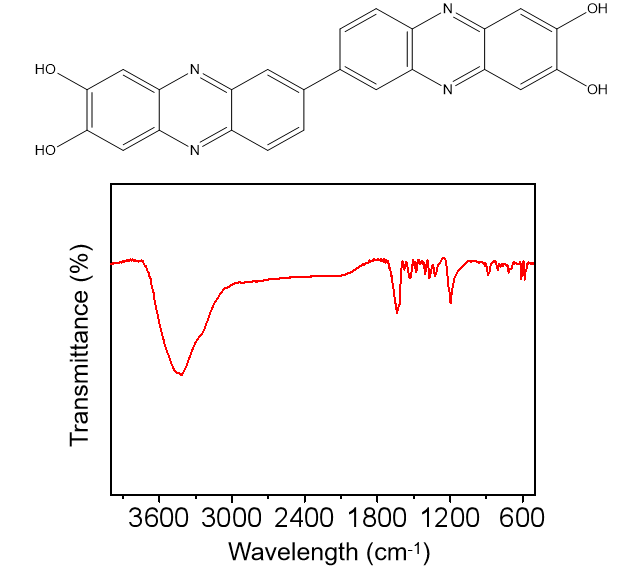


**Figure S6.** FT-IR spectra of BPZT. The peaks at around 3440 cm^-1^ can be assigned to the stretching vibration of O-H bond. Specifically, the peak observed at 1633 cm^-1^ corresponds to the stretching vibration of C=N bonds. The peaks located at 1635, 1577, 1529 and 1480 cm^-1^ can be attributed to C=C bonds in skeleton vibration of aromatic rings. The peak at 1370 cm^-1^ corresponds to the stretching vibration of C-N bond. The peak located at 1200 cm^-1^ matches with stretching vibration of C-O bond. The peaks located at 1042, 880 and 804 cm^-1^ attach to the in-plane and out-of-plane bending vibrations of C-H bond, respectively.


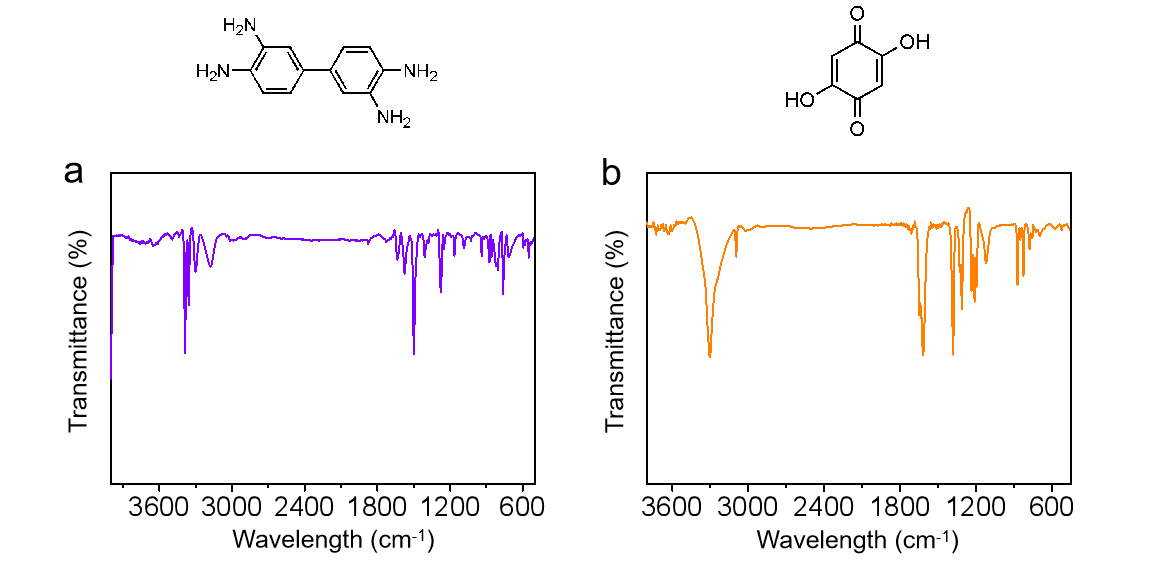


**Figure S7.** FT-IR spectra of raw materials, (a) 3,3'-diaminobenzidine and (b) 2,5-dihydroxybenquinone.

(a) The peaks between 3400 and 3100 cm^-1^ can be attributed to the stretching vibration of N-H and C-H bonds, respectively. The peaks at 1636, 1574, 1497, 1411 cm^-1^ stem skeletal stretching vibrations of benzene rings. The peak located at 1275 cm^-1^ corresponds to C-N bonds. The peaks located at 1250, 808, 762 and 714 cm^-1^ represent the in-plane and out-of-plane bending vibrations of C-H bonds.

(b) The peak at 3302 cm^-1^ belongs to O-H bond. The peaks at 1645 cm^-1^ and 1616 cm^-1^ can be assigned to C=O and C=C bonds, respectively. The peaks at 1380 cm^-1^ can be attributed to the in-plane bending vibration of O-H bonds, respectively. The peaks between 1235, 1210, 1192 and 1120 cm^-1^ correspond to the stretching vibration of C-O and C-C bonds, respectively. The peaks at 1310, 872 and 825 cm^-1^ match with the in-plane and out-of-plane bending vibrations of C-H bonds.


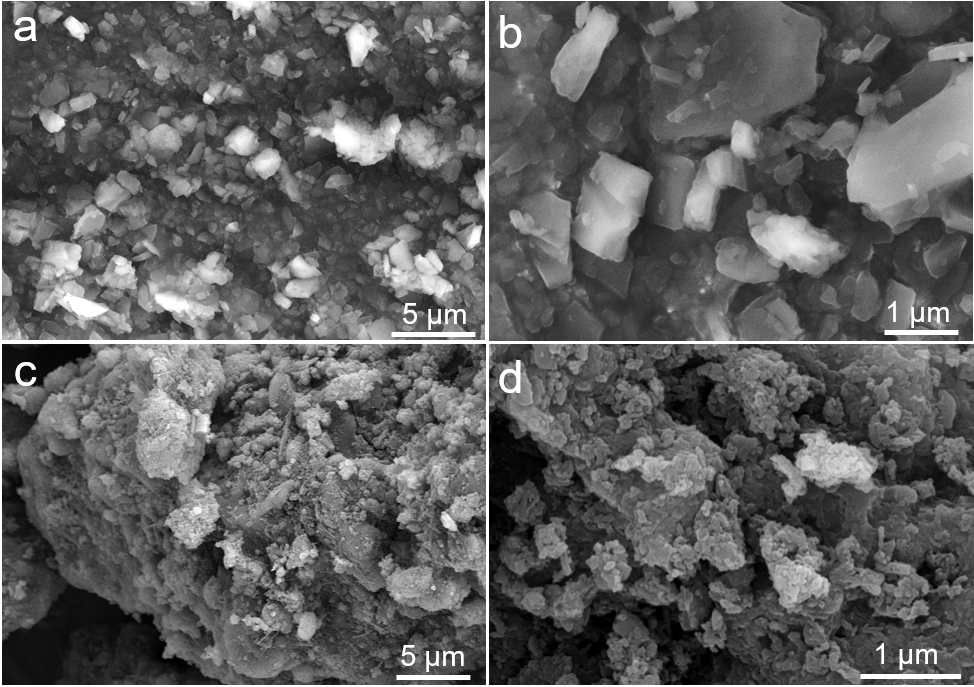


**Figure S8.** SEM images of prepared (a, b) DHP and (c, d) BPZT samples, respectively.


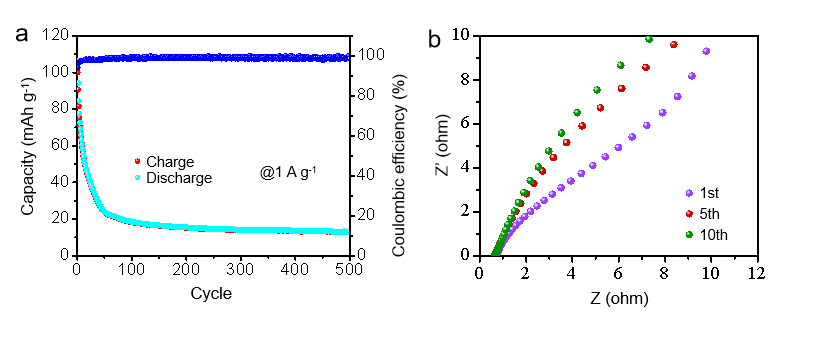


**Figure S9.** The electrochemical property of DHP in 2 M H_2_SO_4_ electrolyte. (a) Cycling stability of DHP at a current density of 1 A g^-1^. (b) Nyquist plots of DHP electrode.


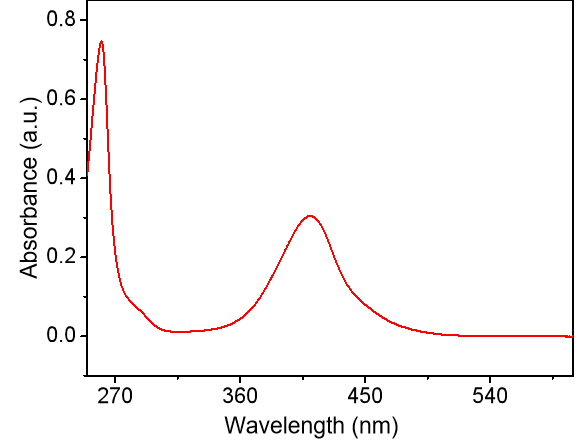


**Figure S10.** UV-Vis spectrum of 0.05 mM DHP in 2 M H_2_SO_4_ solution.


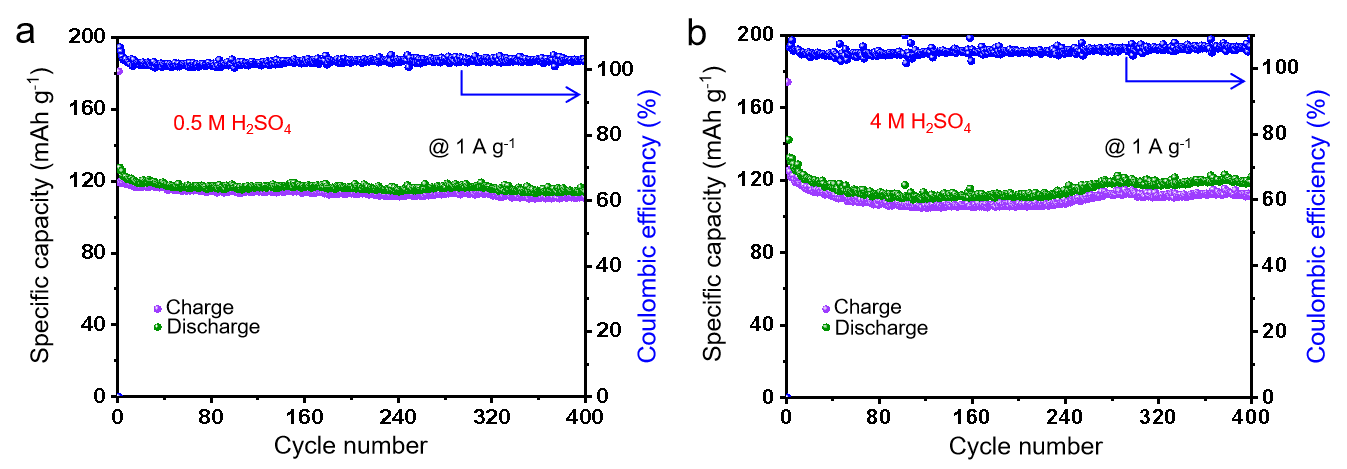


**Figure S11.** Cycling performance of the BPZT electrode in (a) 0.5 M H_2_SO_4_ electrolyte and (b) 4 M H_2_SO_4_ electrolyte at a current density of 1 A g^-1^, respectively, with a mass loading of 2 mg_BPZT_ cm^-2^.


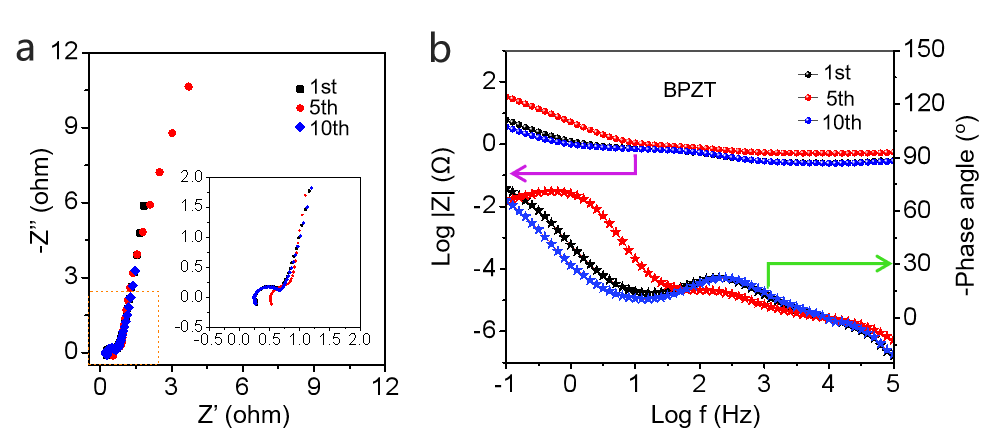


**Figure S12.** Nyquist (a) and Bode plots (b) of BPZT electrode measured in 2 M H_2_SO_4_ electrolyte.

The Nyquist and bode plots of the BPZT electrode in 2 M H_2_SO_4_ electrolyte are provided, as shown in **Figure S12**. From the EIS data, it can be seen that high-frequency intercepts are below 1 ohm, corresponding to low electrolyte and electrical contact resistance. The semicircle diameter of about 0.5 ohm corresponds to low charge transfer resistance. Bode plots of phase angle versus frequency can be used to analyse the relative capacitive and resistive contributions, with ideal capacitance at phase angle of -90^o^, and resistive processes at phase angle of 0^o^.^[S1-S3]^ **Figure S12b** shows the corresponding bode plots of BPZT half battery. The phase angle of between -60^o^ and -70^o^ is observed at the low frequency, where pseudocapacitance is dominant for charge storage. However, the phase angle rapidly decreases with the increase of frequency, which indicates that the resistance contribution rooted from pseudocapacitance dominates.^[S4]^ The Nyquist and bode analyses suggest the low resistance and rapid charge transfer in BPZT half battery, thus conducive to the fast kinetics, which is consistent with the dominant pseudocapacitive and slight diffusion-limited charge storage nature of the BPZT electrode.


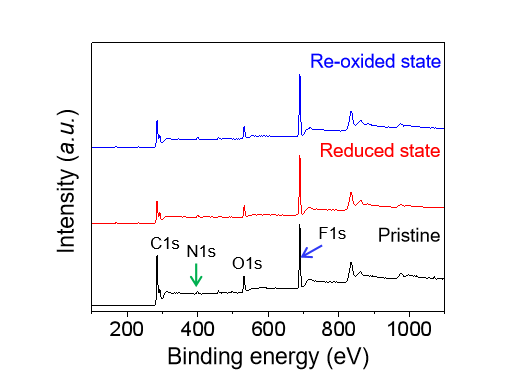


**Figure S13.** The survey XPS spectra on BPZT electrode at pristine, reduced and re-oxided states, respectively.

The characteristic peak of F 1s XPS located at 690.1 eV is related to the PTFE binder.


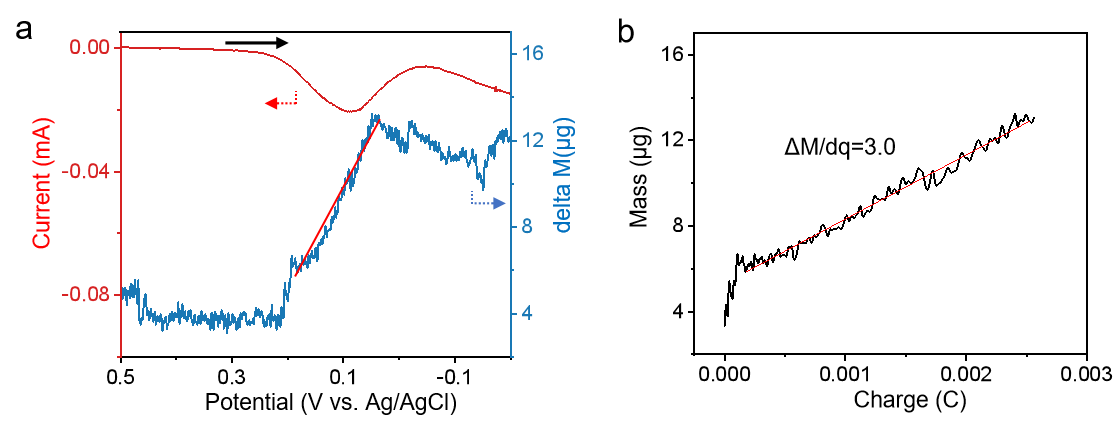


**Figure S14**. (a) Cyclic voltammetry cathodic scan (red) of the BPZT electrode in 2 M H_2_SO_4_ at 1 mV s^-1^, and matched mass change response as recorded by EQCM. (b) Matched mass versus charge curve in the voltage range from 0.5 to -0.2 V for BPZT electrode.


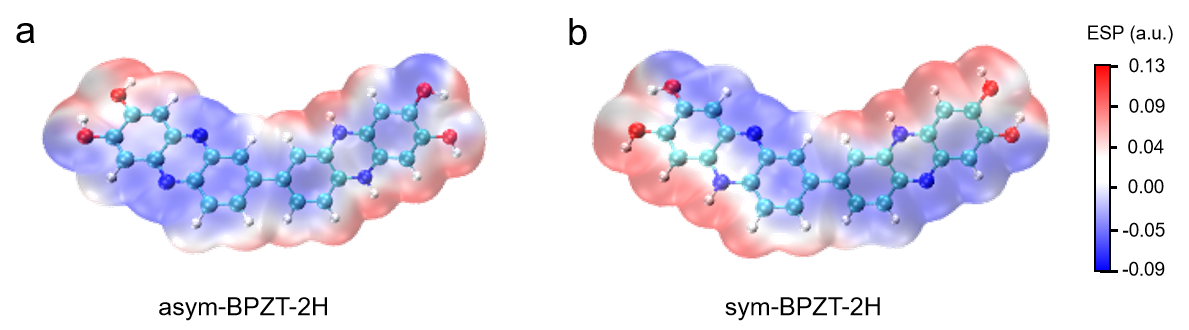


**Figure S15.** Calculated ESP distribution of asym-BPZT-2H and sym-BPZT-2H molecules, with blue and red representing the electron-rich and electron-deficit regions of a molecule, respectively.


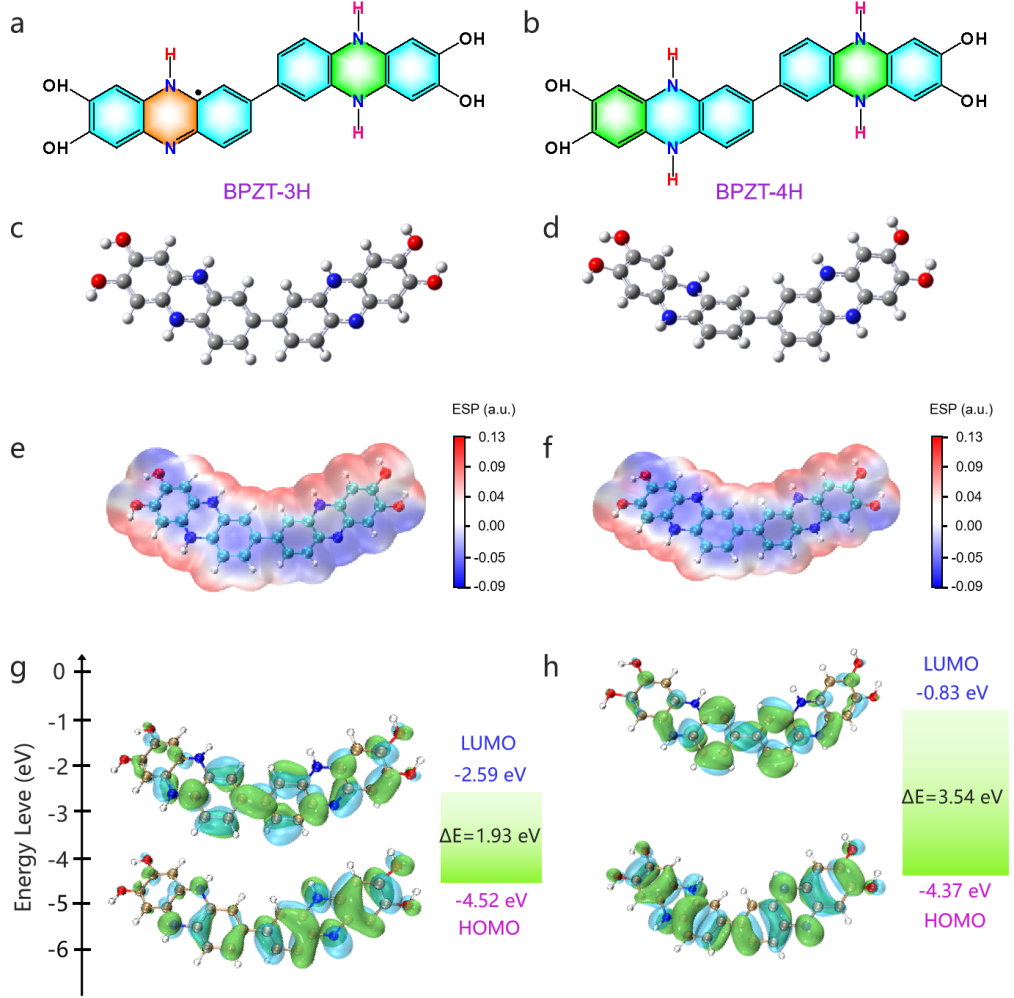


**Figure S16.** The DFT calculation results of BPZT-3H and BPZT-4H based on hypothetical three/four-electron reduction of BPZT molecule. (a, b) The molecular structures of BPZT-3H and BPZT-4H. (c, d) Optimized structures of BPZT-3H and BPZT-4H. (e, f) ESP mapping of BPZT-3H and BPZT-4H molecules. (g, h) LUMO and HOMO energy level of BPZT-3H and BPZT-4H, respectively.

**
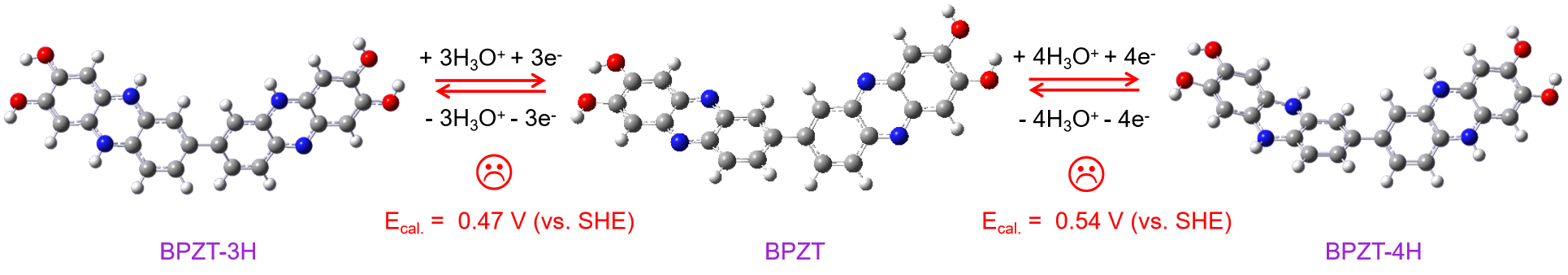
**

**Figure S17.** Schematic diagram of two possible redox reaction pathways of BPZT based on three/four-electron reaction process in a single step.


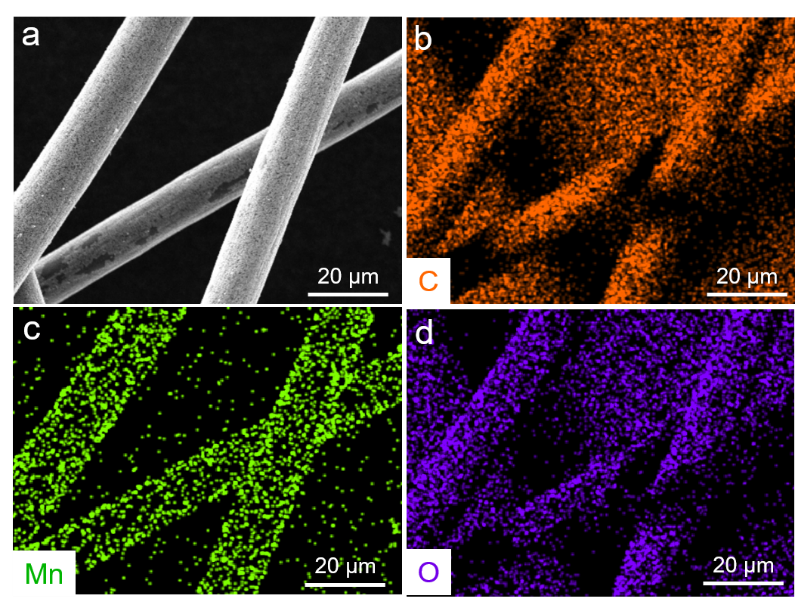


**Figure S18**. SEM images and elemental mapping images with electrodeposited 5 mAh cm^-2^ MnO_2_ on GF electrode.

It is observed that the Mn and O elements are in uniform distribution on GF electrode.


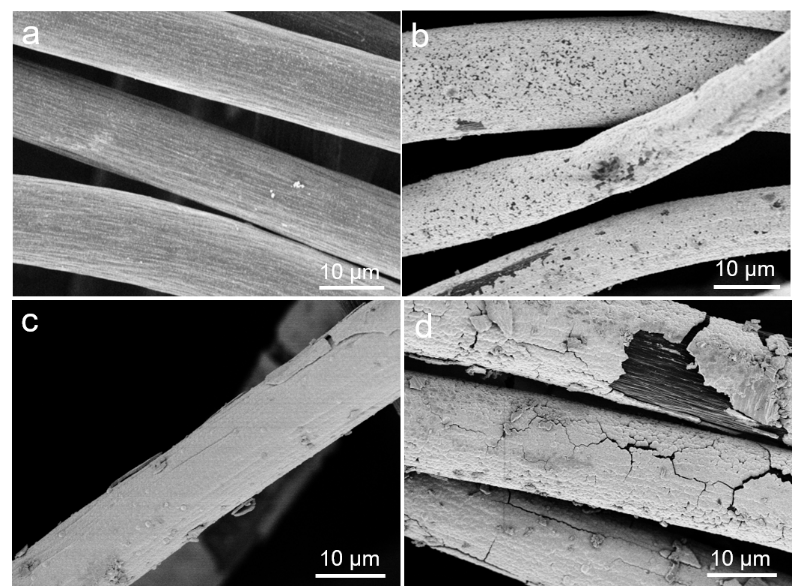


**Figure S19**. SEM images of various electrodeposition amount of MnO_2_ on GF electrode: (a) pristine, (b) 5 mAh cm^-2^, (c) 10 mAh cm^-2^, and (d) 15 mAh cm^-2^.

The deposited MnO_2_ begins to fall off from GF electrode when the deposition amount exceeds 15 mAh cm^-2^.

**
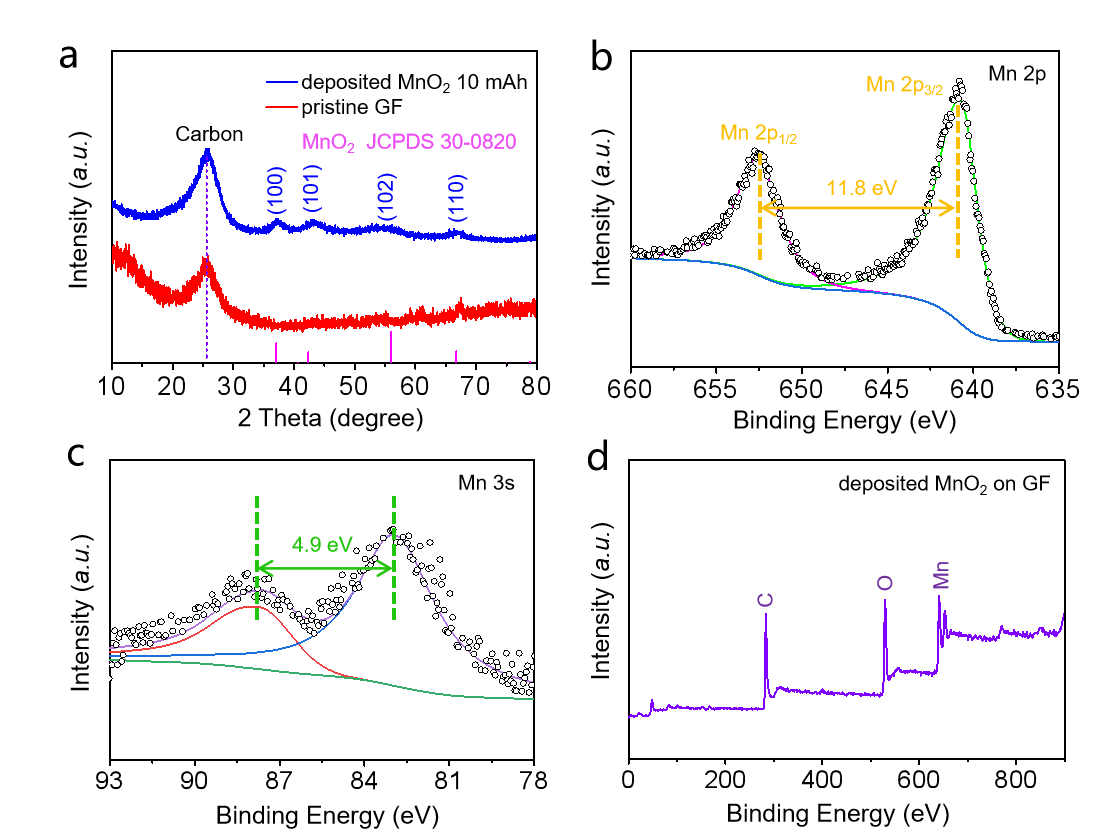
**

**Figure S20**. (a) XRD pattern of MnO_2_@GF with 10 mAh deposition capacity and pristine GF electrode. (b-d) XPS spectra of MnO_2_@GF cathode: (b) Mn 2p, (c) Mn 3s, (d) Survey XPS spectra.


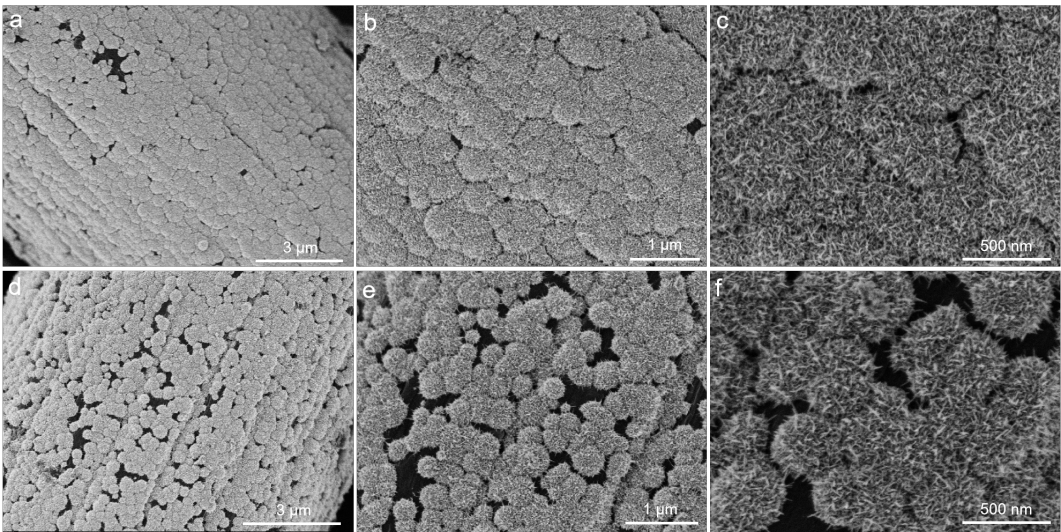


**Figure S21**. SEM images of MnO_2_@GF cathode at oxidized state (a-c) and reduced states (d-f) after 200 cycles in MnO_2_@GF//BPZT full cell.

There is no apparent change in the surface morphology of MnO_2_@GF in the redox process over 200 cycles.


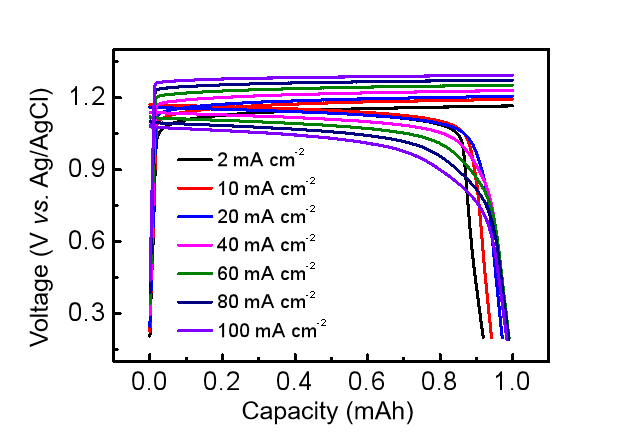


**Figure S22**. GCD profiles of MnO_2_@GF with 1 mAh deposition capacity at different current densities ranging from 2 mA cm^-2^ to 100 mA cm^-2^.

As current densities increasing, the potential gap in deposition/dissolution process gradually enlarged, while the Coulombic efficiency improved.


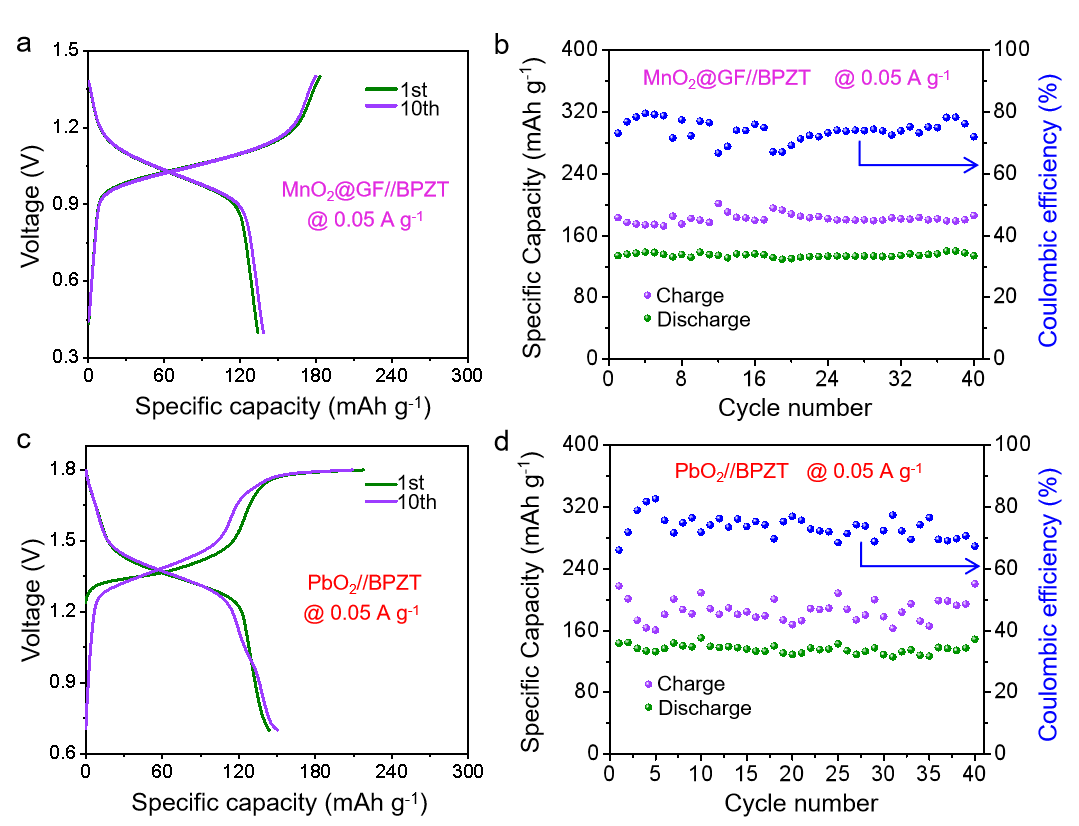


**Figure S23**. GCD curves and cycling performance of MnO_2_@GF//BPZT battery in the hybrid electrolyte of 2 M H_2_SO_4_ + 2 M MnSO_4_ at a low current density of 0.05 A g^-1^.


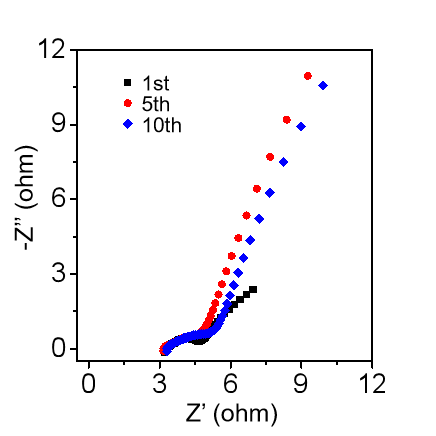


**Figure S24**. Nyquist plots of MnO_2_@GF//BPZT full battery in 2 M H_2_SO_4_ + 2 M MnSO_4_.


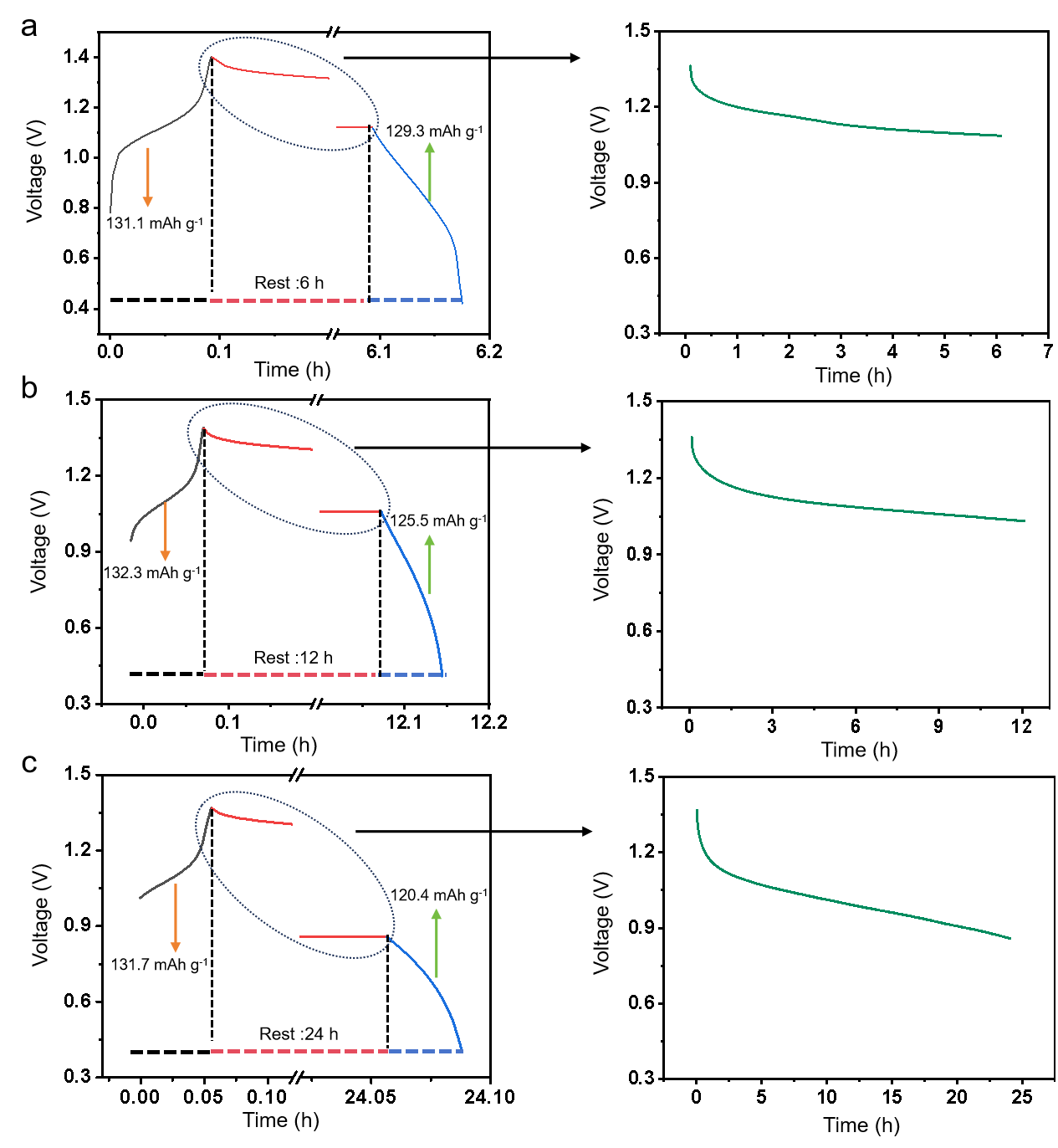


**Figure S25**. Electrochemical stability of the MnO_2_@GF//BPZT full battery surveyed by self-discharge experiments, with the anodic mass loading of 2 mg_BPZT_ cm^‑2^.


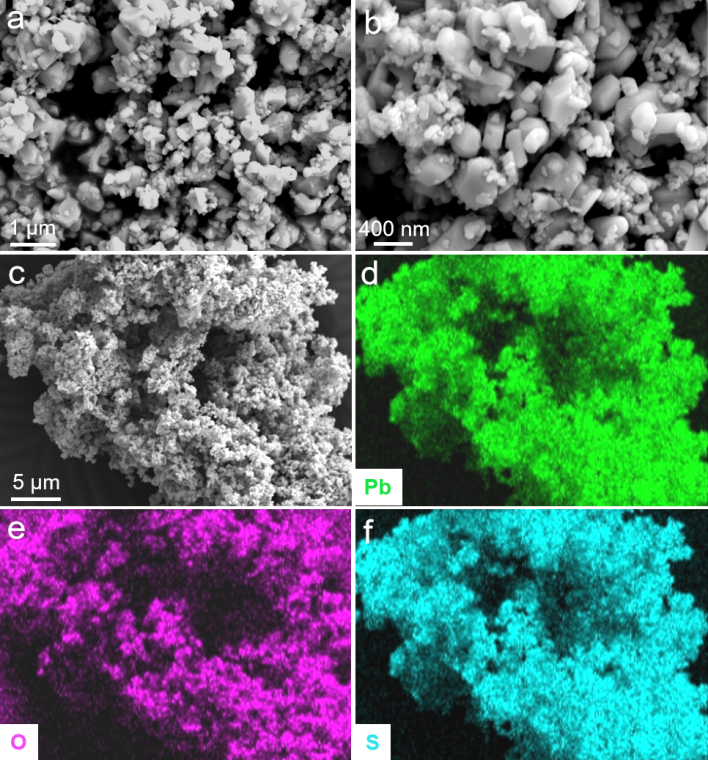


**Figure S26**. SEM images of PbO_2_ cathode.


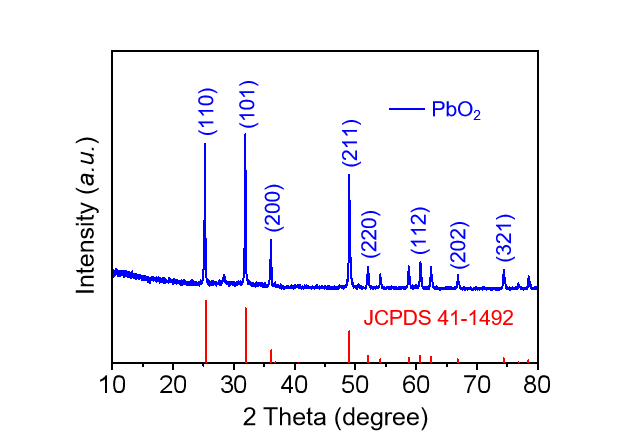


**Figure S27**. XRD pattern of PbO_2_ cathode.

**
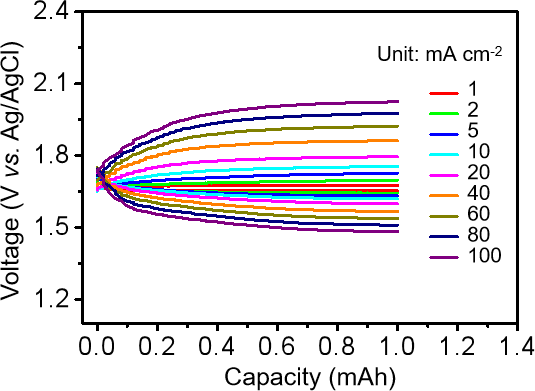
**

**Figure S28**. GCD profiles of PbO_2_ with 1 mAh deposition capacity at different current densities ranging from 1 mA cm^-2^ to 100 mA cm^-2^.


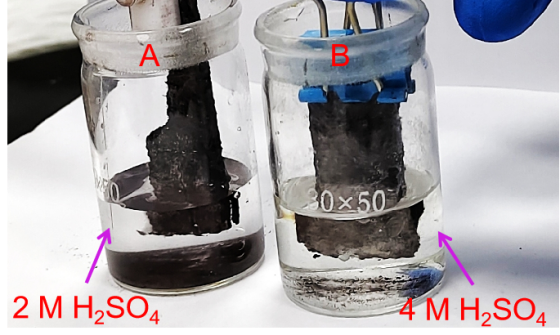


**Figure S29**. Photograph of PbO_2_ electrode immersion in 2 M H_2_SO_4_ and 4 M H_2_SO_4_ electrolytes after 24 h.


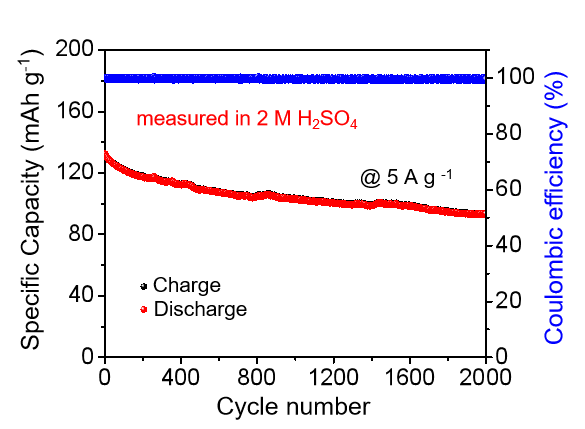


**Figure S30**. Cycling performance of PbO_2_//BPZT full battery in 2 M H_2_SO_4_ at a current density of 5 A g^-1^.


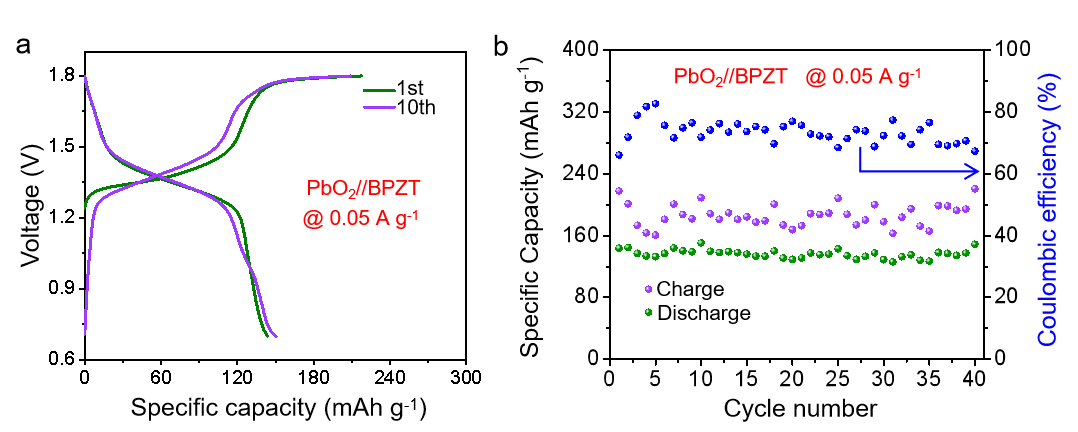


**Figure S31**. GCD curves and cycling performance of PbO_2_//BPZT full battery in 4 M H_2_SO_4_ electrolyte at a low current density of 0.05 A g^-1^.


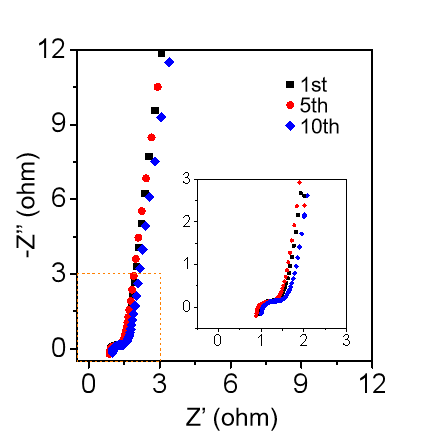


**Figure S32**. Nyquist plots of PbO_2_//BPZT full battery in 4 M H_2_SO_4_.


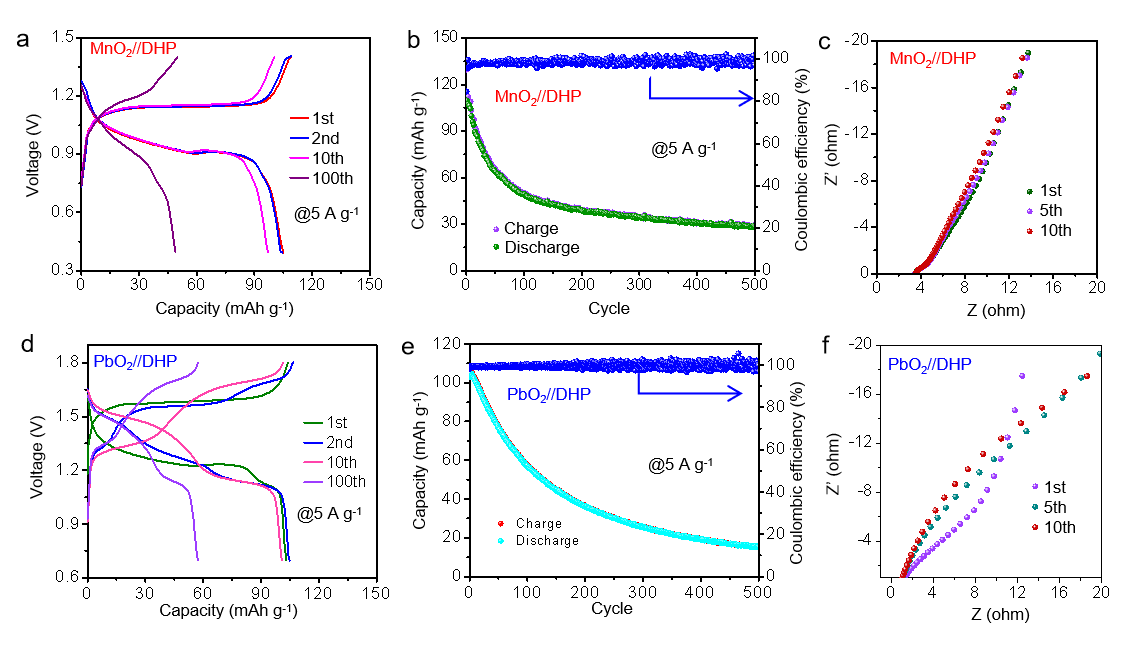


**Figure S33.** (a-c) Electrochemical performance of MnO_2_@GF//DHP full battery measured in a hybrid electrolyte of 2 M H_2_SO_4_ + 2 M MnSO_4_. (a, b) GCD curves at several selected cycles and cycle performance at 5 A g^-1^ for MnO_2_@GF//DHP full battery. (c) Nyquist plots of MnO_2_@GF//DHP full battery. (d-f) Electrochemical performance of PbO_2_//DHP in 4 M H_2_SO_4_ electrolyte. (d, e) GCD curves and cycle performance at 5 A g^-1^ for PbO_2_//DHP full battery. (f) Nyquist plots of PbO_2_//DHP full battery.

**Table S1.** Potential comparison of several organics electrodes in PrIBs.

| **Ref.** | **Organics electrodes** | **Electrolytes** | **Average electrode potentials (V vs. SHE)** |
| --- | --- | --- | --- |
| 8 | HDC (cathode) | 1 M H_2_SO_4_ or quasi-solid electrolyte | 1 |
| 13 | PTO (anode) | 2 M H_2_SO_4_ | 0.48 |
| 22 | PTO (anode) | 4 M H_2_SO_4_ | 0.5 |
| 23 | DTT (anode) | 2 M H_2_SO_4_ | 0.38 |
| 24 | TMBQ (anode) | 0.5 M H_2_SO_4_ | 0.4 |
| S5 | PEDOT-AQ (anode) | 0.1 M 2-fluoropyridinium triflate-2-fluoropyridine in MeCN | ~0.42 |
| S6 | HATN (anode) | 0.05 M H_2_SO_4_ | ~0.2 |
| S7 | ALO (anode) | 2 M HBF_4_ + 2 M Mn(BF_4_)_2_ | 0.27 V |
| S8 | PCHL-rGO (cathode) | 5 M H_2_SO_4_ | ~0.68 V |
| S9 | DHAQ (anode) | 1 M H_2_SO_4_ | 0.19 V |
| S10 | pDTP-NQ (cathode)  pDTP-AQ (anode) | 1 M H_2_SO_4_ | ~0.39 V  ~0.05 V |
| S11 | PNAQ as cathode  PNAQ as anode | 4 M H_2_SO_4_ | 0.81 V  0.2 V |
| **This work** | **BPZT(anode)** | 2 M H_2_SO_4_ | **~0.29 V** |

**Table S2.** Original calculation results employing the implicit solvent model.

| **Species** | **Sum of electronic and thermal Free Energies/Hartree B3LYP-D3(BJ)/6-311G(d,p)** |
| --- | --- |
| BPZT | -1443.116486 |
| sym-BPZT-2H | -1444.288254 |
| asym-BPZT-2H | -1444.306857 |
| BPZT-3H | -1444.892792 |
| BPZT-4H | -1445.49462 |

**Table S3.** Calculation results of implicit CPCM solvent model using 6-311G (d,p) basis set according to **Table S2**.

| **Redox process** | **ΔG (eV)** | **E_(2)_ (V *vs.* SHE)** |
| --- | --- | --- |
| BZPT+2H^+^ + 2e^-^ → sym-BZPT-2H | -9.16 | 0.30 |
| BZPT+2H^+^ + 2e^-^ → asym-BZPT-2H | -9.67 | 0.55 |
| BZPT + 3H^+^ + 3e^-^ → BZPT-3H | -19.27 | 0.47 |
| BZPT + 4H^+^ + 4e^-^ → BZPT-4H | -14.25 | 0.54 |

**Table S4.** Performance comparison of several PrIBs.

| **Ref.** | **Battery systems** | **Cell voltage (V)** | **Specific Capacity**  **(mAh g^-1^)** | **Energy density**  **(Wh Kg^-1^)** | **Mass loading**  **(mg cm^-2^)** | **Cycle life (Cycles) and capacity retention** |
| --- | --- | --- | --- | --- | --- | --- |
| 8 | HDC//activated carbon | 0.3 V | 50 mAh g^-1^  at 0.1 A g^-1^ | - | 3.66 | 2000 at 1 A g^-1^ (89%) |
| 13 | MnO_2_@GF //PTO | 0.86V | 210 mAh g^-1^  at 0.2 C | 132.6 | 4 | 5000 at 2.5 C (80%) |
| 22 | PbO_2_//PTO | 1.25 V | 395 mAh g^-1^  at 0.04 A g^-1^ | 161 | 2.2 | 1500 cycles at 2 C (96%) |
| 23 | MnO_2_@GF//DTT | 0.94 V | 212 mAh g^-1^  at 0.05 A g^-1^ | 185 | 3 | 50000 at 2 A g^-1^ (72%) |
| 24 | MnO_2_//TMBQ | 0.86 V | 300 mAh g^-1^  at 1 C | 166.4 | 1.27 | 4000 at 5 C (77%) |
| S5 | PEDOT-AQ//PEDOT-BQ | 0.5 V | 103 mAh g^-1^  at 0.07 A g^-1^ | 62.5 | 0.25-1.25 | 150 at 0.4 A g^-1^ (79.5%) |
| S6 | MnO_2_//HATN | 1 V | 260 mAh g^-1^  at 0.1 A g^-1^ | 118 | 1.33 | 500 at 1 A g^-1^ (92.65%) |
| S7 | MnO_2_@CF//ALO | 1.2 V | 145.5 mAh g^-1^ at 1 A g^-1^ | 110 | 1-2 | 300 at 5 A g^-1^ (66%) |
| S8 | PCHL-rGO//Pb | 1.06 V | 208 mAh g^-1^  at 0.2 A g^-1^ | - | 1 | 3000 at 10 A g^-1^ (65%) |
| S9 | MnO_2_//DHAQ | 1.1 V | 105 mAh g^-1^  at 0.88 A g^-1^ | - | 3 | 2600 at 1.13 A g^-1^ (60%) |
| S10 | pDTP-NQ//pDTP-AQ | ~0.34 V | 78 mAh g^-1^  at 0.5 A g^-1^ | 35.1 | 1 | 2000 at 1 A g^-1^ (56%) |
| S11 | PNAQ//PNAQ | 0.61 V | 85.3 mAh g^-1^ at 5 C | ~51 | 1~2 | 500 at 50 C (70%) |
| **This work** | **MnO_2_@GF//BPZT** | **1.07 V** | **152 mAh g^-1^**  **at 0.2 A g^-1^** | **125 Wh kg^-1^** | **2** | **20000 at 5 A g_BPZT_^-1^ (90%)** |
|  |  |  |  |  | **10** | **12500 at 5 A g_BPZT_^-1^ (90%)** |
|  | **PbO_2_//BPZT** | **1.44 V** | **156 mAh g^-1^**  **at 0.2 A g^-1^** | **128 Wh kg^-1^** | **2** | **13500 at 5 A g_BPZT_^-1^ (95%)** |
|  |  |  |  |  | **10** | **4200 at 5 A g_BPZT_^-1^ (91%)** |

**References**

[S1] J. Ko, M. Sassin, D. Rolison, J. Long, *Electrochim. Acta*, **2018**, *275*, 225e235.

[S2] N. Chen, J. He, H. Xuan, J. Jin, K. Yu, M. Shi, C. Yan, *Compos. Part B* **2024**, *270*, 111145-111154.

[S3] P. Xiao, N. Chen, J. Liu, L. Yang, D. Chen, M. Shi, *Prog. Org. Coat.* **2014**, *186*, 108017-108025.

[S4] W. Sugimoto, H. Iwata, K. Yokoshima, Y. Murakami, Y. Takasu, *J. Phys. Chem. B* **2005***,* *109*, 7330e7338.

[S5] R. Emanuelsson, M. Sterby, M. Strømme, M. Sjödin, *J. Am. Chem. Soc.* **2017**, *139*, 4828-4834.

[S6] Y. Dai, X. Yan, J. Zhang, C. Wu, Q. Guo, J. Luo, M. Hu, J. Yang, *Electrochim. Acta.* **2023**, *442*, 141870.

[S7] T. Sun, H. Du, S. Zheng, J. Shi, Z. Tao, *Adv. Funct. Mater.* **2021**, *31*, 2010127.

[S8] F. Yue, Z. Tie, S. Deng, S. Wang, M. Yang, Z. Niu, *Angew. Chem., Int. Ed.* **2021**, *60*, 13882-13886.

[S9] J. Yu, J. Li, Z. Y. Leong, D. S. Li, J. Lu, Q. Wang, H. Y. Yang, *Mater. Today Energy.* **2021**, *22*, 100872.

[S10] X. Wang, J. Zhou, W. Tang, *Energy Storage Mater.* **2021**, *36*, 1-9.

[S11] T. Sun, H. Du, S. Zheng, J. Shi, X. Yuan, L. Li, Z. Tao, *Small Methods.* **2021**, *5*, 2100367.
